# Identification of recurrent regulated alternative splicing events across human solid tumors

**DOI:** 10.1093/nar/gkv210

**Published:** 2015-04-23

**Authors:** Miri Danan-Gotthold, Regina Golan-Gerstl, Eli Eisenberg, Keren Meir, Rotem Karni, Erez Y. Levanon

**Affiliations:** 1 Mina and Everard Goodman Faculty of Life Sciences, Bar-Ilan University, Ramat Gan 52900, Israel; 2 Department of Biochemistry and Molecular Biology, the Institute for Medical Research Israel-Canada, Hebrew University-Hadassah Medical School, Ein Karem, 91120 Jerusalem, Israel; 3 Raymond and Beverly Sackler School of Physics and Astronomy and Sagol School of Neuroscience, Tel Aviv University, Tel Aviv 69978, Israel; 4 Department of Pathology, Hadassah Medical Center, Hebrew University, Jerusalem, Israel

## Abstract

Cancer is a complex disease that involves aberrant gene expression regulation. Discriminating the modified expression patterns driving tumor biology from the many that have no or little contribution is important for understanding cancer molecular basis. Recurrent deregulation patterns observed in multiple cancer types are enriched for such driver events. Here, we studied splicing alterations in hundreds of matched tumor and normal RNA-seq samples of eight solid cancer types. We found hundreds of cassette exons for which splicing was altered in multiple cancer types and identified a set of highly frequent altered splicing events. Specific splicing regulators, including RBFOX2, MBNL1/2 and QKI, appear to account for many splicing alteration events in multiple cancer types. Together, our results provide a first global analysis of regulated splicing alterations in cancer and identify common events with a potential causative role in solid tumor development.

## INTRODUCTION

Alternative splicing (AS), the process by which multiple distinct mRNAs are formed from a single gene, is a major source of protein diversity in humans. Current estimations, based on genome-wide approaches, suggest that more than 90% of human genes undergo alternative splicing (1,2). AS may alter the function of a given protein in various ways, including the production of protein variants with opposite biological functions (3).

Alternative splicing has been implicated in cancer. Many key proteins associated with tumor biology including proteins with roles in apoptosis, cell cycle regulation, invasion and metastasis undergo cancer-associated alternative splicing (4–6). In recent years, genome-wide approaches significantly extended the number of annotated AS events altered in cancer, and allowed the discovery of pathways and programs that are differentially regulated in cancer cells (6–12). In many of these high throughput studies, a significant alteration results from aberrant expression and regulation of splicing factors. These RNA binding proteins target and specify exon inclusion or exclusion by binding to splicing enhancer or silencer sequences on the pre-mRNA, in proximity to or within the alternative exon. For example, *RBFOX2* is downregulated in ovary and breast cancers, and dictates many changes in the alternative splicing pattern of these cancers (7,12). Polypyrimidine tract binding protein (*PTB/PTBP1*) is overexpressed in ovarian cancer and gliomas, and has been shown to promote invasive behavior through splicing pattern changes in genes related to cell migration (13–16). *SRSF1* (*SF2*/*ASF*) can function as a proto-oncogene, and its overexpression results in transformation of cell lines. Moreover, it alters the splicing pattern of cancer-related splicing isoforms of genes associated with cell motility and proliferation (17,18). As the splicing factors associated with cancer may have competing effects on the splicing of specific exons, the ultimate splicing pattern in cancer is complex and hard to predict (6,19,20). However, there are specific splice variants that are preferred by cancer cells, and many of them were shown to have altered splicing patterns in several tumor types as well as a functional role in cancer development. For example, the genes *MacroH2A, IGFR, BIN1, PKM, MKNK2, S6K1* and *TNC* have been verified to be differentially spliced in cancer, and to have an important role in tumor initiation and progression (17,21–32).

Most of the approaches used for global identification of cancer-associated splicing events, based on high-throughput reverse transcriptase-polymerase chain reaction (RT-PCR) systems, microarrays and high throughput sequencing, were limited to a pre-defined set of splice variants. In addition, each of these studies mainly focused on a single cancer type. Furthermore, the number of normal and tumor samples in most of the studies was small, limiting the strength of these analyses. To our knowledge, only a few studies compared altered splicing patterns across different cancer types. These studies found common altered splicing patterns and regulation between two or three cancer types (7,33).

Here, we performed a systematic analysis of 343 matched tumors comprising eight cancer types, and normal tissues to characterize alternative splicing alterations, and identified splice variants that were preferred by several cancer types. Using de-novo identification of altered cassette exons, we identified 1188 significantly altered splicing events, 430 (36%) of which were significantly changed in more than one cancer type. Most of these common splicing events changed in the same direction (either exclusion or inclusion in tumor versus normal), though some were altered in opposite directions, mainly when comparing renal clear cell carcinoma with other types of cancers. Several of the splicing events that showed a very high rate of alteration in the same direction either in different cancer types or within the same cancer were validated in matched tumor and corresponding normal tissue taken from various sources; the vast majority of the splicing events changed in tumor versus normal tissue according to the prediction from our analysis of the TCGA (The Cancer Genome Atlas) data. In order to identify splicing factors regulating cancer-associated splicing events, we performed sequence analysis followed by expression profiling, and found RBFOX2, QKI, PTBP1, CELF2 and MBNL1/2 splicing activities are strongly associated with many of the altered splicing events in several cancer types examined.

## MATERIALS AND METHODS

### Data preprocessing

TCGA RNA-seq data for eight cancer types (breast invasive carcinoma (BRCA), colon adenocarcinoma (COAD), kidney renal clear cell carcinoma (KIRC), liver hepatocellular carcinoma (LIHC), lung adenocarcinoma (LUAD), prostate adenocarcinoma (PRAD), head and neck squamous cell carcinoma (HNSC) and thyroid carcinoma (THCA)) were downloaded from TCGA data portal as bam files (34). For uniform alignment parameters, each bam file was converted back to a Fastq file. Quality estimation was performed using the Fastqc program. Fastq files that failed in the ‘Per sequence quality scores’ or the ‘Per base sequence quality’ tests were removed from downstream analyses (these tests will fail if (i) the most frequently observed mean quality of the reads is <20; or (ii) the lower quartile of the quality at any base in the reads is less than 5 or (iii) if the median for any base is <20).

### Reads alignment

STAR aligner (version 2.3.0) was used to align each file uniquely to the Hg19 human genome (35). We kept only uniquely aligned reads, with a minimum splice junction overhang of five nucleotides (default parameters except outFilterMultimapNmax 1, outSJfilterCountUniqueMin 10 2 2 2, outSJfilterCountTotalMin 10 2 2 2, alignSJDBoverhangMin 5). The vast majority of the junctions identified (68 998, 99.8%) were canonical junctions (dinucleotides GT and AG for donor and acceptor sites, respectively), the others were non-canonical GC-AG (128, 0.2%).

### Identification of altered splicing patterns

**Figure 1.**
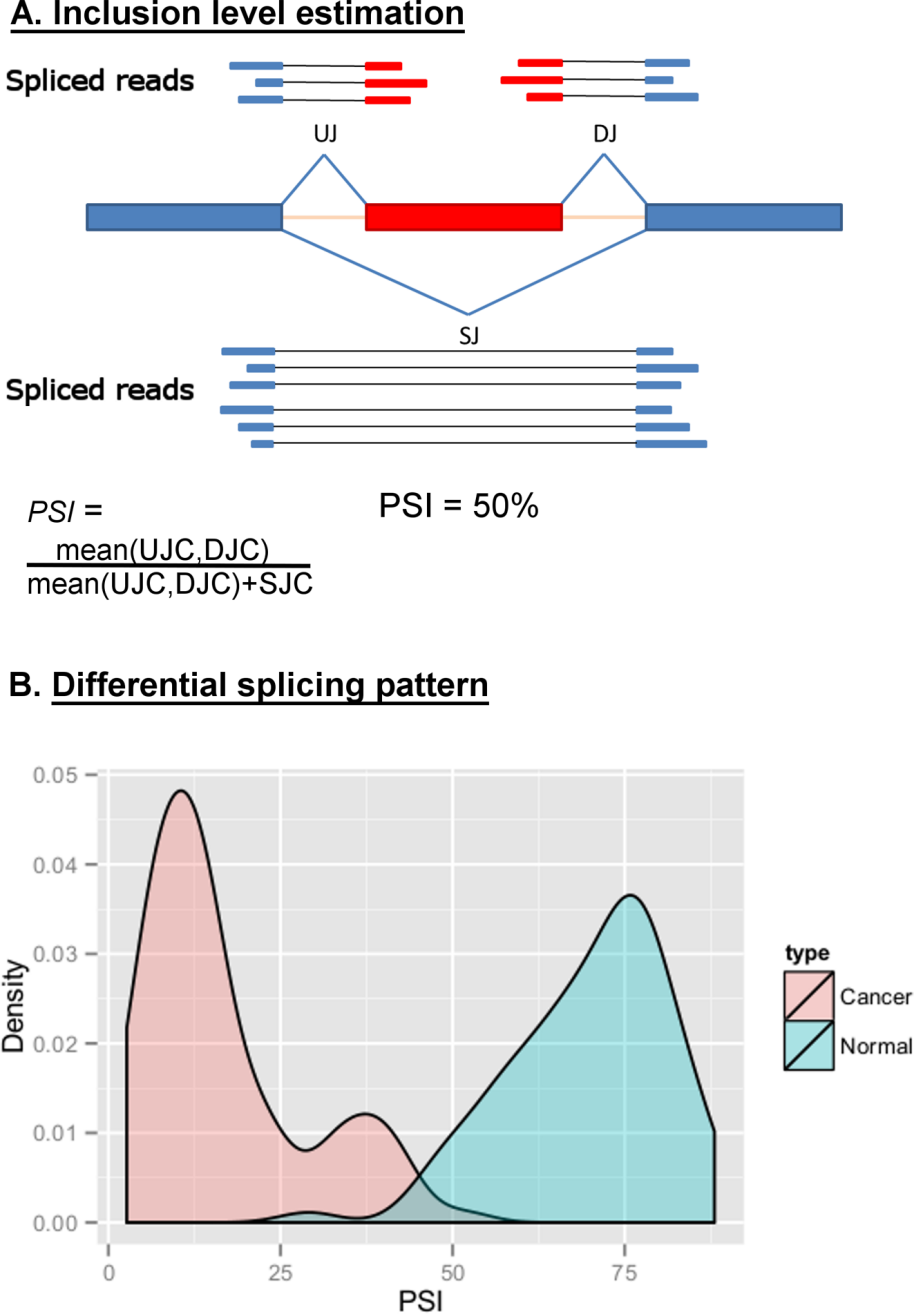
Identification of cancer-associated splicing events. (**A**) Reads that were gap-aligned to the genome were used to infer cassette exons.Estimation of the inclusion level (PSI) based on the number of reads supporting the exon–exon junctions that define the splicing event: UJ (upstream junction) and DJ (downstream junction) – reads supporting inner junctions, SJ (skipped junction) – reads supporting skipping junction. UJC, DJC and SJC are upstream, downstream, skipped junction read count, respectively. (**B**) PSI was calculated for each splicing event in all normal and tumor samples. Statistically significant changes between paired samples (tumor-normal) from the same individual are defined as cancer-associated splicing events. FBLN2 exon PSI distribution in breast tumors and normal samples is shown.

The STAR SJ output file, which summarizes all split-mapping reads supporting exon-exon junctions, was used to identify altered splicing patterns. Cassette exons were defined by three junctions; two of them supporting exon inclusion, and an outer junction with the same boundaries (upstream junction (UJ) and downstream junction (DJ) and skipped junction (SJ), respectively. see Figure 1). In order to exclude detection of false positives, we required each junction to be supported by a minimum of five reads in at least one sample of the dataset, and the inner junctions (UJC and DJC, upstream and downstream junction counts) to have similar reads counts in all the samples of the dataset ((|UJC − DJC| < 10 or |UJC − DJC| < 2 × min(UJC,DJC))).

Exon inclusion level (PSI, percent spliced-in) was calculated for each predicted cassette exon based on the number of reads supporting UJ, DJ and SJ junctions (Figure 1A). In order to include only reliable events and accurate PSI levels, only cassette exons with all three junctions supported by at least 10 reads (0.5(UJC + DJC) + SJC ≥ 10) in each of at least 15 matched samples of the each cancer type dataset were analyzed (highly expressed exons). Wilcoxon paired test was used to infer significant change in the PSI value between matched normal and tumor samples from the same individuals. For each cancer type, differentially expressed cassette exons were only called as such if both of the following conditions were fulfilled (i) false detection rate (FDR) of 0.05, (ii) at least 10% difference in the average PSI level (average |ΔPSI| ≥ 10%) between matched normal and tumor samples.

In order to evaluate the conservation of these exons, mouse alternative cassette exons were obtained from the UCSC database and aligned to the Hg19 human genome using the liftOver utility (minimum 10% unique alignment, total of 10 413 cassette exons aligned, 88% of all UCSC annotated mouse cassette exons). Mammalian conserved alternative exons were obtained from Supplementary Table S4 of (36). Overlap of these cassette exons with the cancer altered cassette exons was determined using Bedtools (37).

### Bioinformatic analyses

The statistical analysis was done using R (the R Project for Statistical Computing (http://www.r-project.org/)). All bioinformatics analyses details are provided in the Supplementary Methods.

### RT-PCR analysis of matched normal and tumor samples

Matched normal and tumor samples were obtained from the Hadassah Medical Center tissue bank, under institutional Helsinki ethical approval. RNA was extracted from the frozen tissues using metal beads in TRI reagent (Sigma) in a bullet blender at 4°C for 5 min. Total RNA (500 ng) was reverse transcribed using M-MLV reverse transcriptase (Promega). PCR was performed on 1/50 (2 μl) of the cDNA, in 25 μl reactions containing KAPA2G Fast HotStart DNA Polymerase in a ×2 ReadyMix format with loading dye (Kapa Biosystems), 0.2 mM of each primer and 8% (v/v) DMSO. PCR conditions were 95°C for 5 min, then 34 cycles of 94°C for 15 s, 60°C for 15 s and 72°C for 45 s, followed by 10 min at 72°C. PCR products were separated on 1.5% or 2% agarose gels. Normalization was performed using β-actin. Primers are listed in Supplementary Table S11.

## RESULTS

### Identification of altered alternative splicing in eight cancer types

In order to identify frequent and recurrent changes in the splicing patterns of tumors compared with their corresponding normal tissue, we examined poly-A selected RNA-seq data from eight cancer types from TCGA (34): BRCA (38), COAD (39), KIRC (40), LIHC, LUAD (41), PRAD, HNSC and THCA. Using split-mapping reads, we inferred *de-novo* cassette exons that were alternatively spliced in each RNA-seq sample. A skipping event was defined by three inferred exon–exon junctions; two of them supporting exon inclusion and an outer junction with the same boundaries (UJ and DJ and SJ, respectively; Figure 1) (see ‘Materials and Methods’ section). In order to obtain accurate PSI levels and to avoid false positive detection of splicing events, we analyzed only predicted cassette exons that had at least 10 reads supporting exon inclusion or exclusion in each of at least 15 matched samples. Matched samples were used to enable identification of altered events that are affected by factors unrelated to the cancer state, i.e. age, and to attain higher accuracy results. A total of 47 969 highly expressed skipping events comprising of 46 681 cassette exons (several exons have more than two possible neighbors) were analyzed (Supplementary Table S1). Exon inclusion level (PSI) was calculated for each of these skipping events (see ‘Materials and Methods’ section). Correlation of PSI values in normal samples was very high (Pearson correlation >0.95 for all normal samples). This high correlation rate is comparable with the high correlation obtained for RNA-seq expression experiments (42).

We found 1188 skipping events to be significantly altered (absolute average PSI change ≥ 10%, FDR = 0.05 using paired Wilcoxon test) in at least one cancer type (these splicing events included 1173 cassette exons in 860 annotated genes, Supplementary Figure S1, Supplementary Table S2). The vast majority of these exons, 1038, were previously annotated, either as a single exon or multiple consecutive exons, in either the UCSC (928 exons) or Ensembl (1029 exons) gene databases. Most of the UCSC annotated exons (80%, 741 exons) were defined as cassette exons in the UCSC database. These results strongly support the mapping quality and validate the filtering processes performed.

Since exon inclusion and global gene expression levels may be connected by cellular mechanisms such as non-sense mediated decay (NMD), which can reduce the total expression of a transcript with a premature termination codon, we further checked the correlation between the gene expression level and the exon PSI level. In most cases (more than 70%), the correlation was weak (correlation coefficient < 0.3), indicating that expression level is probably not the cause for the altered splicing patterns observed in these cases (Supplementary Table S2).

Reassuringly, 43 exons from these cancer-associated cassette exons were previously identified and validated as significantly changed in cancer cells (Supplementary Table S3 and refs. therein). Among these 43, we found splicing events in the transcripts encoding IGFR, BIN1, RAC1 and TNC, which were extensively studied and found to exhibit a role in either tumorigenesis or cancer progression (22,24,30,43). Moreover, 84 of the genes encoding the cancer-associated exons were identified in a previous high throughput RT-PCR-based study of breast cancer altered splicing (7), and, as expected, most of these 84 genes (65 of them) were found here to encode breast cancer-associated cassette exons.

Almost half of the cancer-associated cassette exons (46.29%, 543 cassette exons) are alternatively spliced in mouse as well, and 128 of them overlapped a small subset of cassette exons defined by Merkin *et al*. as ‘broadly alternative’ exons which are alternatively spliced in mammals (hypergeometric *P*-value for the mammals conserved exons overlap with cancer associated exons < 1e−90) (36). Consistent with being conserved across species, most of the identified cancer-regulated exons preserved the reading frame (67.5% in the cancer associated cassette exons versus 46.6% in the examined highly expressed cassette exons, Chi-square *P*-value < 2.2e−16), as previously observed in orthologous exons alternatively spliced in more than one organism (44). This significant fraction of conserved alternatively spliced cassette exons suggests most of the cancer regulated exons are functional and tightly regulated.

### Common cassette exons altered in different cancer types

**Figure 2.**
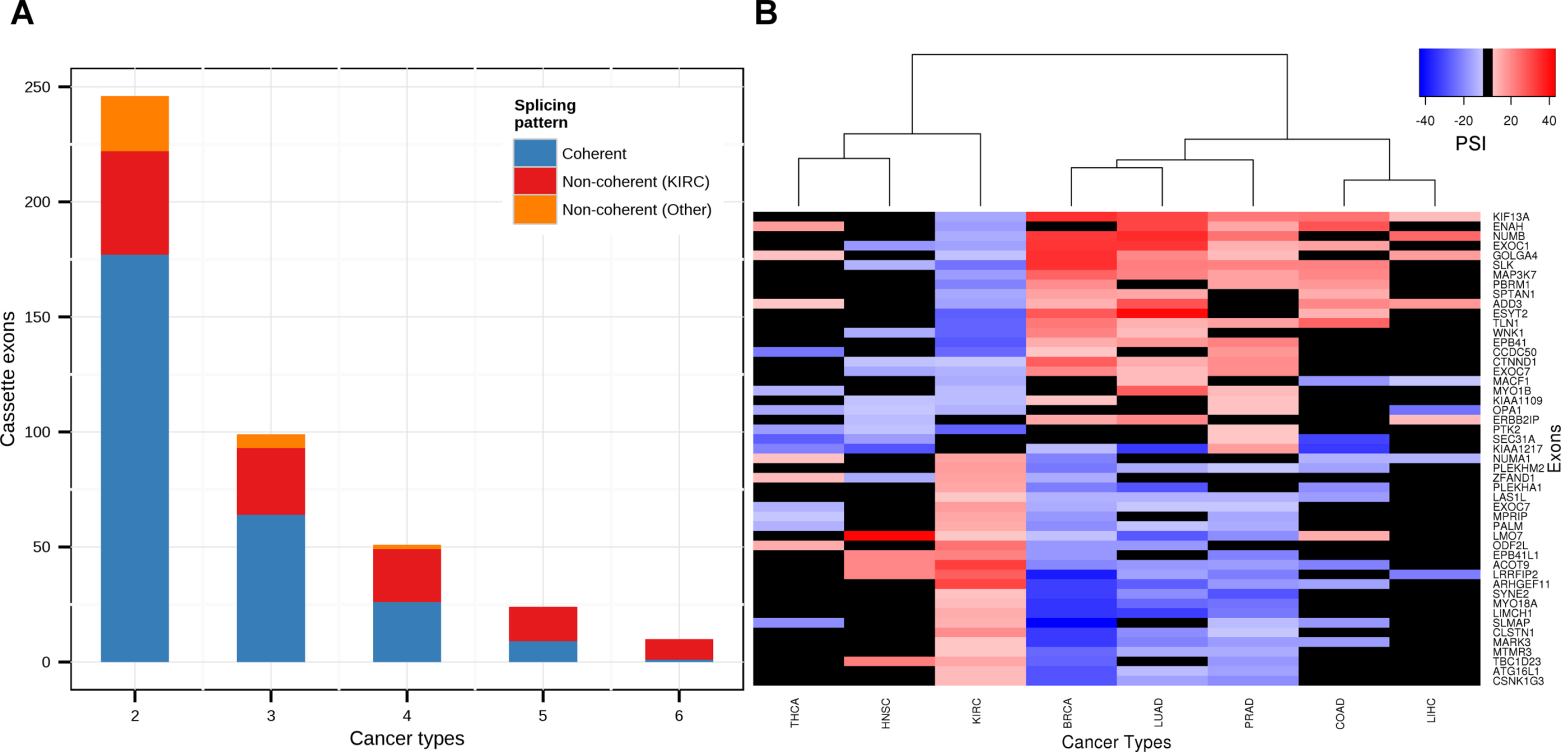
Common splicing events shared in different cancer types. (**A**) Bar plot showing the number of common splicing events significantly altered in 2–6 tumor types, blue denotes the number of coherent events, red denotes the number of non-coherent events altered in KIRC, orange denotes the number of non-coherent events altered in other cancer types. (**B**) Heatmap of mean PSI value changes (matched tumor – normal) for non-coherent differential alternative splicing events common to at least four cancer types. Black square denotes no significant alteration was observed for the specified exon in the specified cancer type.

Notably, many of the exons detected in previous studies were re-detected here, but in a different cancer type (Supplementary Table S3). Our global analysis reveals that this observation is very common; more than one-third of the splicing events detected had a significant splicing pattern change in more than one cancer type (430 out of 1188). This subset of exons had a higher rate of conserved exons, and 272 of them (63%, Chi-square *P*-value < 1e−15) were found to be alternatively spliced in the mouse. However, 154 cassette exons changed in a ‘non-coherent’ manner, meaning the direction of the change was not consistent across different cancer types. In most of these non-coherent splicing events, the change observed in the KIRC samples was in an opposite direction versus other cancer types, mainly breast, prostate and lung (BRCA, PRAD and LUAD) samples (Figure 2). Interestingly, out of 49 events that were altered in a non-coherent manner in more than three cancer types, 13 exons were previously found to discriminate between mesenchymal and epithelial cell types (out of 41 exons) (45). None of the events that were altered coherently in more than three cancer types (36 events) were found to discriminate between mesenchymal and epithelial cell types. This may either support previous studies suggesting that these changes reflect the change in the composition of the tissue (more mesenchymal or epithelial cells) or a true epithelial to mesenchymal (EMT) or mesenchymal to epithelial transition (MET) in these types of tumor (46,47). The mesenchymal marker Vimentin (VIM) expression levels also support a reverse change between BRCA, PRAD, COAD, LUAD and KIRC (Supplementary Figure S2).

**Figure 3.**
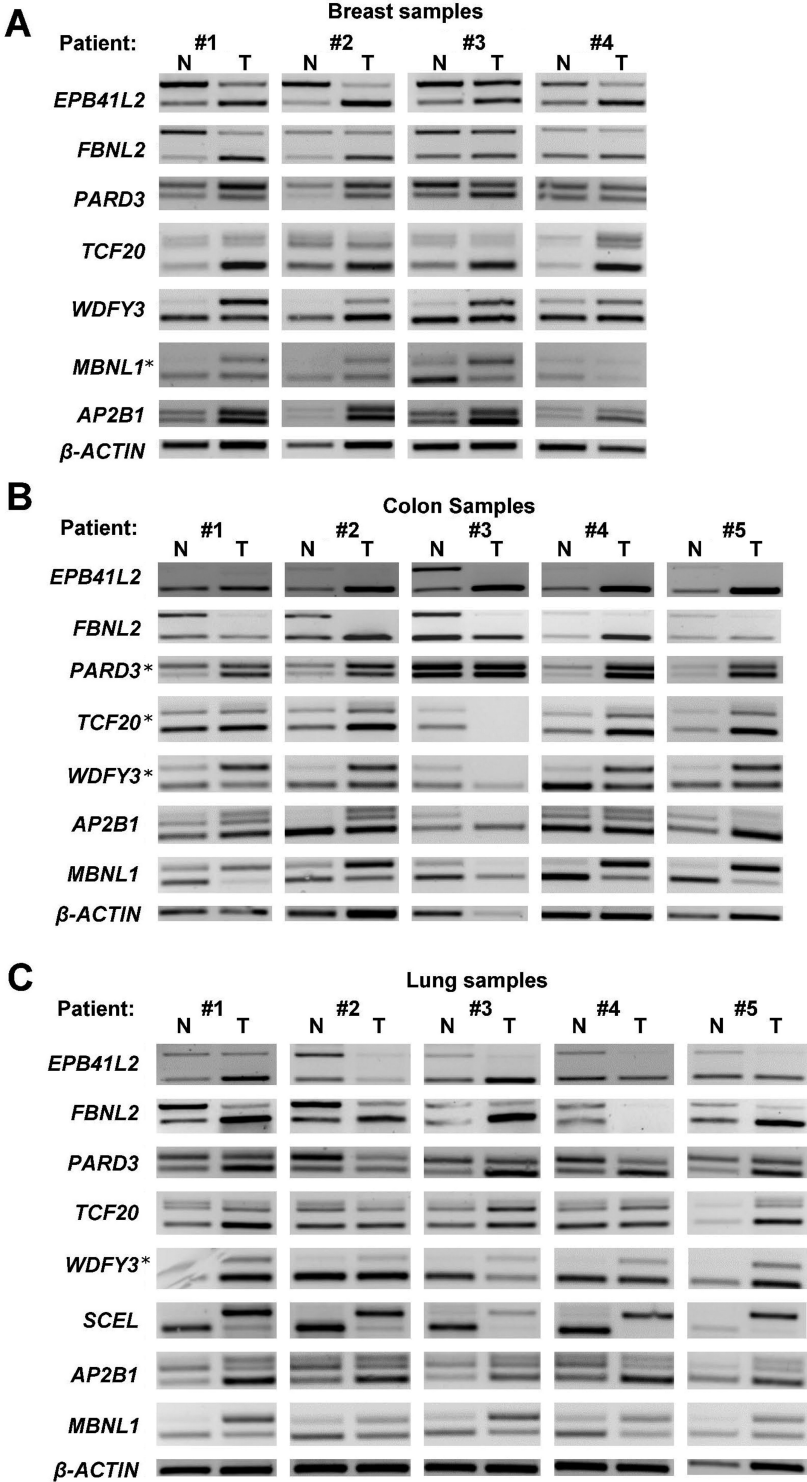
Validation of recurrent alternative splicing changes across patients from three types of cancer. Altered splicing events for genes shown in the figure were examined by RT-PCR analysis. (**A**) Matched breast tumors and the corresponding normal breast tissue of four breast cancer patients; (**B**) matched colon tumors and the corresponding normal colon tissue of five colon cancer patients and (**C**) matched lung tumors and the corresponding normal lung tissue of five lung cancer patients. β-Actin expression is shown as a control for sample recovery and loading. Upper PCR bands, exon inclusion; lower bands, exon exclusion products. Splicing events that were examined in additional cancer type (other than the predicted) are indicated by an asterisk. The predicted pattern of change was observed in most of the samples examined (see Supplementary Table S4 for summary).

The preference of a specific splice variant by several cancer types may indicate that the function of this splice variant contributes to the malignant phenotype (6). Interestingly, in our set, one cassette exon was changed significantly in the same direction in six different cancer types, and nine cassette exon changes were common to five cancer types (Table 1/ Supplementary Figure S3). Seven of these cassette exons preserve the reading frame and the other three are part of the untranslated region, and thus, the resulting transcripts are likely translated to create functional proteins that have different regulation in cancer either at the protein level or the transcript level. To validate these altered splicing events, we performed RT-PCR of matched normal and tumor samples from BRCA, LUAD and COAD patients. Out of five tested events, all five displayed the expected altered splicing pattern in most of the examined samples of each cancer type (Figure 3, Supplementary Table S4).

**Table 1.** Splicing events altered in the same direction in at least five cancer types

Gene name	Cancer types altered significantly	Type of change in cancer	Preservation of the reading frame
*AP2B1*	LIHC, BRCA, LUAD,COAD, HNSC	Exclusion	+
*DEF8*	BRCA, LUAD, KIRC, HNSC, COAD	Exclusion	− (5′ UTR)
*EHBP1*	LIHC, BRCA, LUAD, COAD, THCA	Inclusion	+
*EPB41L2*	BRCA, LUAD, KIRC, COAD, THCA	Exclusion	+ (2 exons)
*FN1* (EDB+)	LIHC, BRCA, LUAD, KIRC, HNSC, THCA	Inclusion	+
*PARD3*	LIHC, BRCA, LUAD, KIRC, HNSC	Exclusion	+
*RPS24*	LIHC,BRCA, LUAD, HNSC, COAD	Exclusion	− (3′ UTR)
*VPS29*	LIHC,BRCA, LUAD, KIRC, THCA	Inclusion	+
*FBLN2*	BRCA, LUAD, HNSC, COAD, THCA	Exclusion	+
*TCF20*	LIHC, BRCA, LUAD, KIRC, THCA	Exclusion	− (3′ UTR)

The EDB exon of the *FN1* gene, whose inclusion was preferred in six different cancer types, was previously found to be highly expressed in HNSC, COAD and LIHC. However, to our knowledge, although it is highly conserved (∼95% amino acid conservation among 22 different vertebrates), no significant function has been assigned to its gene product to date (48–50).

**Figure 4.**
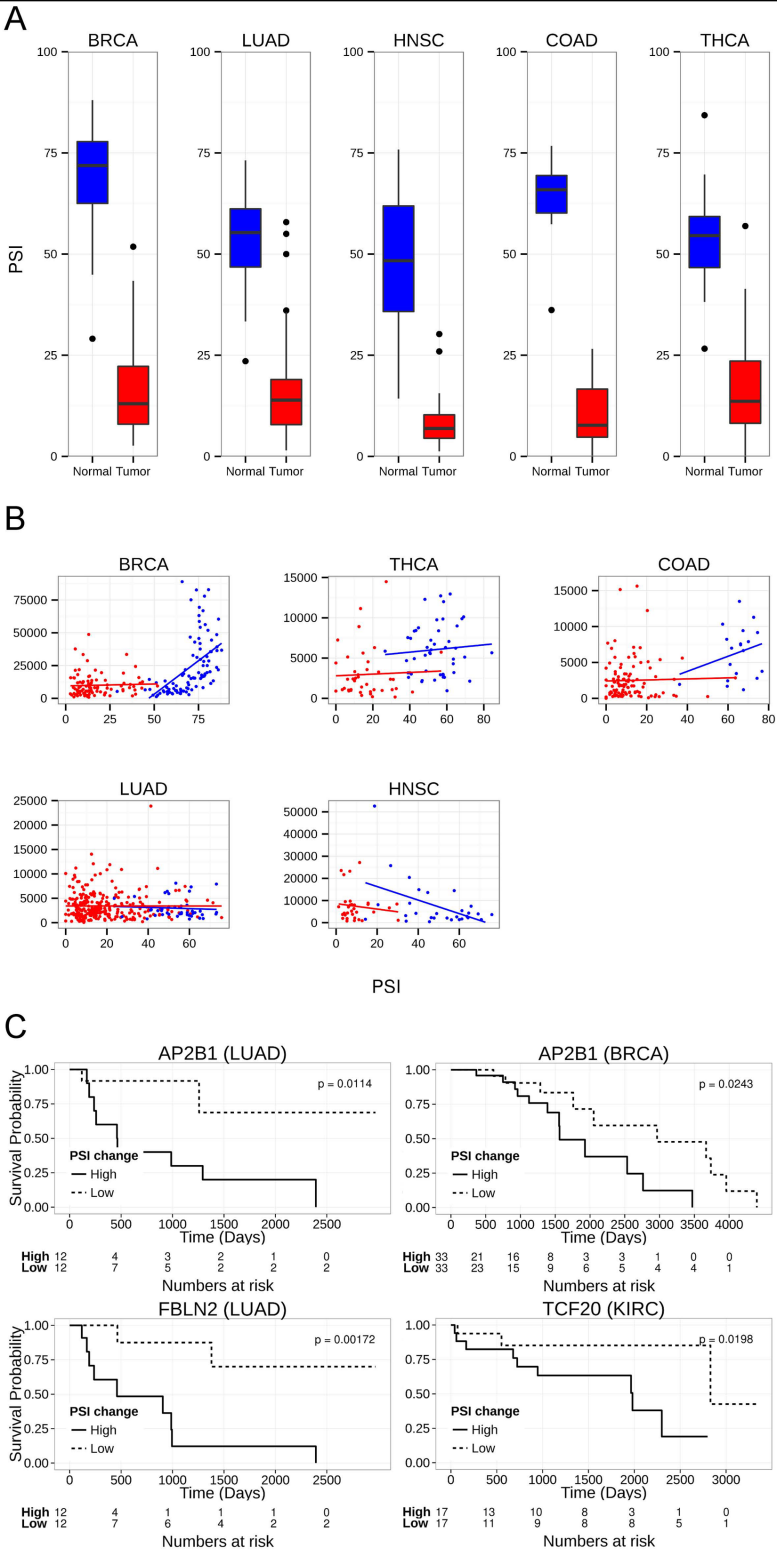
Common similarly altered exons in five cancer types. (**A**) Box plot of PSI levels of FBLN2 in tumor and normal samples in five cancer types. (**B**) Scatter plot of FBLN2 PSI levels versus the normalized counts calculated using the DEseq package (90) in five cancer types. Red dots denote tumor samples, blue dots denote normal samples. (**C**) Kaplan–Meier curve of splicing events altered in at least five tumor types that showed statistically significant change between groups of lower and higher inclusion changes (see ‘Materials and Methods’ section); *AP2B1* in BRCA dataset, *AP2B1* in LUAD dataset, *FBLN2* in LUAD dataset and *TCF20* in KIRC dataset.

Another interesting case is Fibulin-2 (*FBLN2*), an extracellular matrix glycoprotein which is frequently methylated and downregulated in many cancer types (51–54). We found exon 9 was preferentially excluded in five different cancer types (Figure 4A). This exon skipping event was previously observed in LUAD and nasopharyngeal carcinoma. The *FBLN2* short isoform was suggested to drive malignant progression in LUAD; however, in nasopharyngeal cancer, opposite results were reported (55,56). Since *FBLN2* expression changes significantly in many cancer types, we further examined the association of the *FBLN2* splicing pattern with its expression levels. The *FBLN2* exon splicing pattern was significantly correlated with the gene expression level in BRCA, COAD and THCA tumor types; however, no statistically significant correlation was observed for LUAD and HNSC samples (Figure 4B). These results may indicate that the regulation of *FBLN2* expression and splicing pattern are probably not related.

Since we expected these positively selected variants may help promote tumor survival, we further examined the correlation of patient survival with their PSI change. A statistically significant correlation was observed for *AP2B1, TCF20* and *FBLN2* in one or two of the cancer types examined (Figure 4C). In all of these events, higher changes in PSI levels predicted lower survival probability, as expected.

As opposed to breast, colon and thyroid adenocarcinoma, no significant expression change was observed for *FBLN2* in lung adenocarcinoma and it is the only cancer type in which a significant correlation of PSI change and cancer survival was observed. Hence, it may be proposed that although a significant change in splicing was observed for BRCA, COAD and THCA, the reduced gene expression of *FBLN2* probably has a greater effect on the cancer phenotype in these cancers. Taken together, *FBLN2* results in lung adenocarcinoma support an important role for this gene alternative splicing, as previously suggested (55).

Moreover, examination of the exons altered significantly in four cancer types revealed two exons that were studied previously and found to have roles in tumorigenesis and tumor progression. The prevalent isoform of the tumor suppressor *BIN1* in cancer, which includes exon 12A, causes the protein to lose its tumor suppressor activity (24), and the large isoform of tenesin-C (*TNC*) induces cell migration and was suggested to protect cancer cells from immune surveillance (57,58).

As three of the proteins mentioned above are secreted extracellular proteins, we further examined, using the String database, if they interact with one another (59). Indeed, *FN1* interact with both *FBLN2* and *TNC*. This may indicate that the cancer associated variants of these extracellular matrix proteins influence the same properties of the extracellular matrix.

*FBLN2, BIN1, FN1* and *TNC* examples suggest that the variants that are altered in the same direction in several cancer types have significant roles in tumor initiation and progression. Together, these results suggest that similar splicing programs are regulated in different cancer types.

### Cancer splicing markers

Splicing events that give advantage to a specific cancer type tend to recur in many patients. Hence, the identification of a pronounced and consistent splicing event in a single cancer type may indicate its importance to the cancer phenotype. To find such events, we filtered for exons whose splicing pattern was changed consistently (|ΔPSI| ≥ 10%) in more than 90% of the examined matched samples (in which the variant is highly expressed) in each cancer type. This resulted in 31 cassette exons with altered splicing in the vast majority of the tumors compared with the corresponding normal samples (Table 2). In most of these events, the mean PSI change is > 25%, which marks them as good candidates for biomarkers for the related tumor type. Indeed, exon 9 inclusion of the NUMB gene was previously found to be highly prevalent in NSCLC (10). Notably, two cassette exons that were altered significantly in more than four cancer types were also found among these consistently altered cassette exons; exon 9 of the gene *FBLN2*, and the EDB exon of the gene FN1. This further supports their role in the cancer phenotype and their preference by a high percentage of tumors regardless of the cancer type. Moreover, two splicing factors, *MBNL1* and *PTBP2*, were also found to be consistently altered in THCA and COAD respectively, suggesting a feedback loop of the splicing regulation.

**Table 2.** Potential splicing markers

Cancer type	Gene name	#match samples examined	Average PSI change	% samples changed
BRCA	FBLN2	95	−53%	99%
BRCA	ABI3BP	53	−44%	100%
BRCA	WDFY3	21	42%	100%
KIRC	GOLGB1	30	−28%	93%
KIRC	SLC28A1	16	−27%	94%
KIRC	EPB41	50	−28%	92%
KIRC	ARHGEF11	48	−31%	91%
KIRC	CCDC50	61	−25%	90%
KIRC	SYK	46	47%	91%
THCA	PTBP2	22	−29%	95%
THCA	FBLN2	35	−36%	97%
HNSC	LMO7	22	42%	90%
HNSC	FN1	25	25%	92%
HNSC	FBLN2	27	−40%	100%
COAD	ATP2B4	15	−52%	100%
COAD	EHF	15	−23%	93%
COAD	PPP1R13B	17	−25%	94%
COAD	CD44	18	46%	100%
COAD	FBLN2	15	−53%	93%
COAD	ASAP2	18	−28%	94%
COAD	SMTN	18	31%	94%
COAD	LRRFIP2	18	−41%	94%
COAD	MBNL1	17	37%	100%
COAD	EPB41L2	17	−33%	100%
COAD	FLNA	18	34%	94%
LIHC	USO1	26	−44%	96%
LUAD	PTS	36	24%	92%
LUAD	SCEL	28	80%	100%
LUAD	NUMB	36	36%	94%
LUAD	FN1	36	19%	92%
LUAD	VEGFA	36	−30%	97%
LUAD	MYO6	36	−33%	100%
LUAD	ESYT2	36	41%	97%
LUAD	CEACAM1	27	−41%	93%
LUAD	LIMCH1	23	−32%	91%

Splicing events with at least 10% PSI change in least 90% of the samples in the cancer type (in which they are highly expressed) are defined as potential splicing markers.

Cancer types are defined in Table 1.

We validated three additional specific splicing events found in BRCA, LUAD and COAD cancer types using RT-PCR. Thus, in total six splicing markers were validated (splicing events encoded by the genes *FBLN2* and *WDFY3* for the BRCA dataset, *MBNL1, EPB41L2* and *FBLN2* for the COAD dataset and *SCEL* for the LUAD dataset, Figure 3 and Supplementary Table S4). All of the examined events changed in the same direction in the vast majority of the tissues tested as determined by our RNA-seq analysis. The high validation rate by this independent method and independent sample set further supports these results, although only a small number of matched samples for each cancer type were used.

### Gene function analysis of cancer associated splice variants

Previous studies have shown that in many cases of differential splicing events, the genes involved are related, and are part of a large network that is controlled by specific splicing factors (9,60,61). In order to identify related functionality of the differentially spliced genes, we applied network-based analysis for each cancer type. High enrichment of genes related to cytoskeletal organization, cell–cell adhesion and cell movement was observed in most of the examined cancer types (Supplementary Figure S4 and Table S5). Moreover, as expected, genes related to cancer were also enriched in most of the cancer types. In all of the cancer types examined, we found enrichment of genes related to ‘microtubule dynamics’ and ‘formation of cellular protrusions’ (FDR < 0.05, Supplementary Figure S4). Similar functional enrichment was demonstrated in previous studies for BRCA, ovary and COAD cancer types (7,8). Hence, our results support a similar role for alternative splicing in the vast majority of the examined tumor types.

### Identification of regulatory motifs

**Figure 5.**
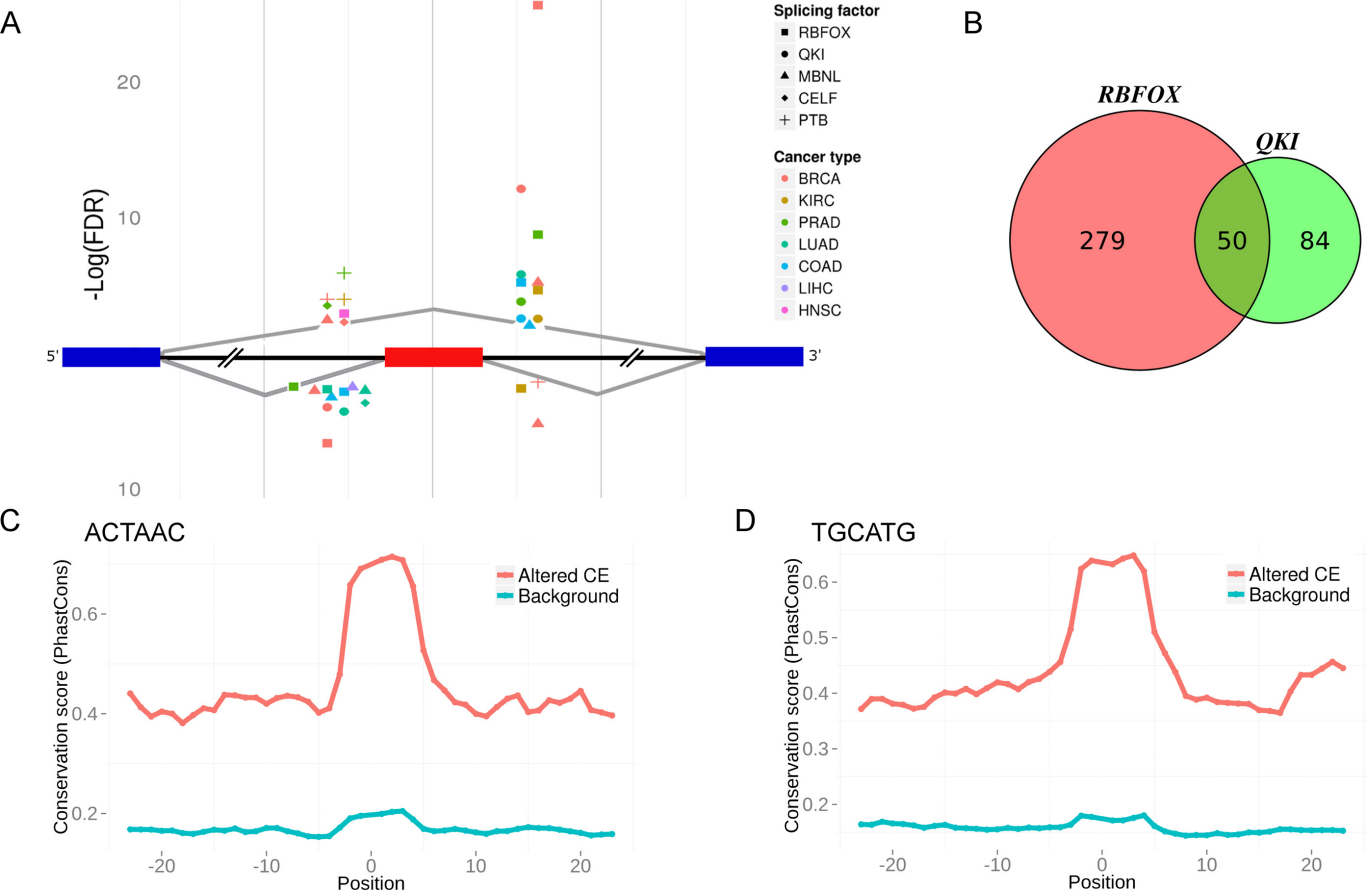
Motif enrichment and conservation in introns flanking cancer altered cassette exons in several cancer types. (**A**) Splicing factor binding motifs were enriched upstream and downstream of the cancer associated exon. The *P*-values were calculated using the hypergeometric test. Enriched motifs were found within 250 nt flanking the exon. In case of several motifs associated with a certain splicing factor, the FDR value presented is the lowest (most significant) one. Small differences in the x-axis within the upstream and downstream introns are intended for symbol clarity only. (**B**) Venn-diagram of the exons whose flanking introns contain QKI and RBFOX motifs. (**C** and **D**) Mean conservation (PhastCons) score versus motif position of (C) ACTAAC motif, and (D) TGCATG motif. Position 0 indicates the middle position of the motif. Red line denotes conservation mean for cassette exons altered in cancer that include the motif within their flanking introns (altered CE), blue line denotes conservation mean of highly expressed cassette exons that were not altered in cancer but include the motif in their flanking introns (Background).

Splicing factors are known to control the inclusion or exclusion of large subsets of exons (7,45,60,62). The high number of splicing event alterations and the common altered splicing events between different cancer types suggest altered activity of splicing factors. To find common splicing factor regulation, we searched for regulatory elements in the sequences within and around the altered exons. We applied a naïve approach and searched for enrichment of k-mers (4–6 nt) within each altered cassette exon and in the adjacent 250 nt of the flanking introns. Since many splicing factors regulate differently exons found upstream or downstream to their binding motifs, the flanking introns analysis was divided to exons that are either included or excluded in each cancer type. No splicing factor signature was found within the exons. We found a significant enrichment of consensus binding sites of PTB, CELF, RBFOX and MBNL in the upstream introns, and RBFOX, QKI and MBNL in the downstream introns flanking the cancer associated cassette exons in more than one cancer type (FDR = 0.01) (63–71). These splicing factors were related to cancer in previous studies (7,12,16,21,25,46,62). In most of the cancer types, the motif that flanked excluded or included exon was found in the same position relative to the regulated exon, indicating a similar change of expression for the splicing factor in these cancers. The results of the analysis are summarized in Figure 5A, and Supplementary Tables S6–S9. In addition, we found a high overlap between exons that had a RBFOX motif in their flanking introns, and those with a QKI motif in their flanking introns (Figure 5B, hyper-geometric *P*-value = 0.004). This supports a recent study suggesting that QKI and RBFOX regulate common splicing targets (46). Together, these results suggest that common splicing factors regulate many of the altered splicing events in at least three of the cancer types examined.

Conservation analysis of the most enriched motifs, QKI and RBFOX motifs (ACUAAC and UGCAUG) in the downstream introns, revealed these motifs were more conserved in the introns flanking the cassette exons that were altered in cancer relative to the cassette exons that were not altered. Also, the mean conservation score of the motif positions was significantly higher compared with the score of the flanking sequences (Figure 5C and D). The increased conservation at the motif region of the altered cassette exons in cancer suggests a primary role for these splicing factors in regulation of gene expression.

### Splicing factors' expression is altered in several cancer types

Next, we further examined the expression profile of the splicing factors whose motifs were highly enriched in the previous section. For this, we applied DEseq analysis on all tumor and normal RNA-seq samples (including samples without a matched pair, Supplementary Table S1) and determined differential expression of these genes in all the cancer types examined; the differentially expressed genes are summarized in Table 3 and Supplementary Table S10. As expected, an expression change was observed for each member of the group of RNA-binding proteins that was related to the motifs found, namely *RBFOX2, QKI, CELF2, MBNL1, MBNL2* and *PTBP1*.

**Table 3.** Altered expression of RBFOX2 and QKI in different cancer types

Splicing factor	BRCA	COAD	PRAD	LUAD	KIRC
RBFOX2	0.65 (0.007)	0.71 (0.01)	0.74 (0.04)	-	-
QKI	0.46 (6.29e-06)	0.51 (7.13e-05)	0.71 (0.021)	0.41 (1.77e-12)	1.32 (0.036)

Cancer types are defined in Table 1.

Opposite trends of expression changes were observed for QKI, MBNL1 and CELF2 in the KIRC compared to other cancer types (upregulation in KIRC versus downregulation in other cancer type). This may explain part of the inconsistent PSI changes we observed.

Most of the expression changes found were in agreement with the PSI change observed in cancer samples and the relative position of the motif. MBNL1/2, RBFOX2 and QKI were downregulated in most of the cancer types and, as expected, their motifs were enriched in these cancer types upstream to exons with higher exclusion and downstream to exons with higher inclusion in cancer (62,72–75). PTBP1 was upregulated in BRCA and its motif was enriched upstream to exons with lower inclusion and downstream to exons with higher inclusion in cancer (66).Together, these results suggest that cancer associated splicing alterations are, in some of the cancers examined, a result of changes in the expression of splicing factors.

### Correlation between recent RBFOX2 and QKI knockdown experiments and splicing patterns of their targets in tumor samples

**Figure 6.**
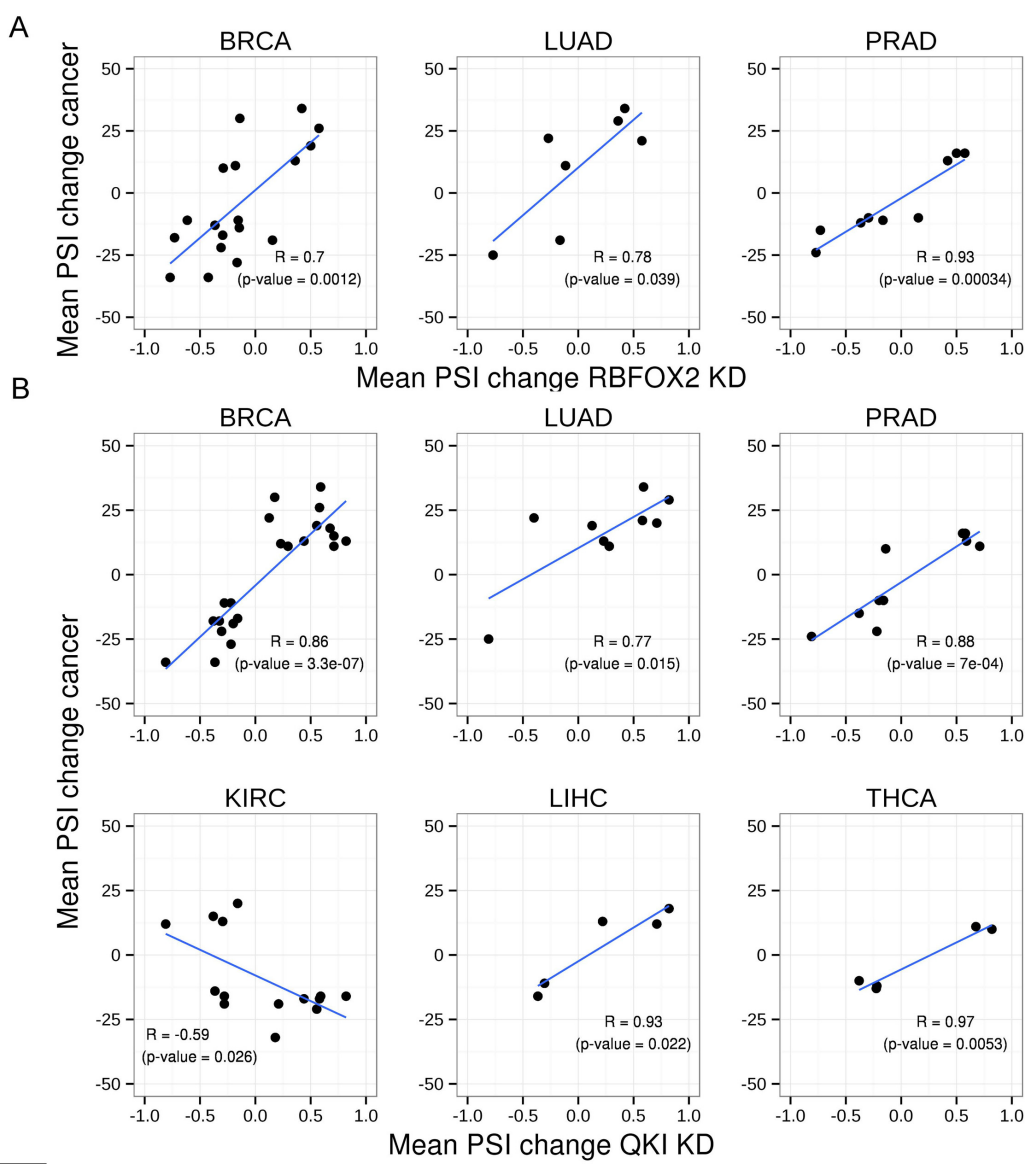
Correlation between RBFOX2 and QKI knockdown experiments and cancer splicing pattern changes. Scatter plot showing mean differences in PSIs of each cancer type of tumor versus normal samples and mean differences in PSIs of (**A**) *RBFOX2* and (**B**) *QKI;* knockdown (46) versus control treatments.

In order to further establish the association of QKI and RBFOX2 with the cancer types examined, we compared the mean PSI changes (all tumors vs. normal samples) in each cancer type with the change in PSI in recently published knockdown and ectopic expression experiments evaluated by either RNA-seq or qPCR (46,62,76). As expected, a strong positive correlation was observed between mean PSI changes in BRCA, LUAD and PRAD and RBFOX2 knockdown PSI changes, while negative correlation was found between mean PSI changes in BRCA and LUAD and the PSI changes in RBFOX2 ectopic expression experiments Figure 6A and Supplementary Figure S5A). No significant correlation was observed in other cancer types.

The same comparison with QKI knockdown revealed statistically significant correlations in all the cancer types examined in the RNA-seq experiment and in five of the cancer types examined in the qPCR experiment (Figure 6B and Supplementary Figure S5B). Positive correlation was observed in BRCA, LUAD, PRAD, COAD, THCA and LIHC, while negative correlation was observed for the KIRC and HNSC.

Since both values compared are derived from average mean values of different tissue samples, tissue/cell types, and state *(in vivo* versus *in vitro*), it is likely that these correlations under represent the actual level of association between these splicing factors and the examined cancer type. Moreover, many of the splicing events examined here are regulated by both QKI and RBFOX2, and a common effect may also affect these associations. Nevertheless, these results further support the association between QKI and RBFOX2 and alternative splicing in the indicated cancers.

## DISCUSSION

We performed a comprehensive analysis of significant and recurrent alternative splicing alterations in eight human cancer types using TCGA RNA-seq data from matched tumor and normal samples. We show that many of these events are shared among different cancer types; our data also suggest that specific splicing factors, namely RBFOX2, QKI, MBNL1/2, PTBP1 and CELF2 are probably responsible for many of the alterations detected in these cancer types.

Using our unbiased approach, no prior knowledge about gene structure was used, we were able to detect >100 previously non-annotated cassette exons. It is likely that these unannotated exons represent true exons as 51% of them have a typical size of one exon (smaller than 250 nt, 69 exons) and of these, the length tends to be a multiple of three (45%, more than the expected 1/3).

Our functional analysis reveals that many of these cancer-associated splicing events are functionally related, and associated with cytoskeletal organization and cell–cell adhesion. This supports a network of splicing variants regulated by specific splicing factors as previously studied in other tissues (60,61). However, it is still not clear which of these events are ‘drivers’, that play a direct role and support cancer initiation and progression, and which events are only ‘passengers’ that have little or no role in cancer biology, and were probably altered by the same splicing factors that affect the drivers. Since we analyzed various cancer types and many samples in each type, we were able to identify frequently recurring events that also exhibited the same pattern of change within and between cancer types. These events are good candidates for actively selected variants that may drive cancer. The results of our analyses revealed a very high frequency of altered splicing in the *FBLN2* and *FN1* genes. These proteins are extracellular proteins that interact with one another and may influence the same extracellular characteristics (77). Thus, the changes we identified in alternative splicing of these genes are appealing potential diagnostic markers for tumor initiation in several cancers, and *FBLN2, AP2B1* and *TCF20* are also good prognostic markers for patient survival in breast, lung, and kidney cancers, respectively (Figure 4). Taken together, it is likely that splicing changes in *FN1, FBLN2, AP2B1* and *TCF20* are also drivers of cancer initiation and progression, which still needs to be determined functionally.

Many splicing events were altered in opposite directions in different cancer types, and most of them exhibited opposite inclusion level changes between KIRC and all the other cancer types. This opposite splicing regulation in KIRC, may result –in part- from the opposite change in level of expression of *QKI, MBNL1* and *CELF2* that we detected in KIRC compared to other cancer types. Moreover, these non-coherent events included many splicing events that are known to differ in epithelial versus mesenchymal cells. Hence, the reason for non-coherent alteration between different cancers may stem from either opposite transition in different cancer types (EMT versus MET) or different composition of the tumor versus the normal tissue as a result of different tissue of origin, as previously suggested (45–47,78). Indeed, EMT transition was found to occur in renal clear cell carcinoma (79,80). It should be noted that although these events are regulated differently in different cancer types, some of them, for example, the *RAC1* variant, were previously shown to have a role in cancer transformation and progression (43,81).

Identification of alternative splicing patterns requires a higher level of expression than identification of differentially expressed genes, and in many cases, one of the variants analyzed is expressed at a low level. Hence, many of the events previously identified using qRT-PCR, which is a more sensitive method (7), were not detected here. Our statistical analysis is highly influenced by the number of matched samples used, and thus, a greater number of significantly altered splicing events are expected as the number of samples increases. Thus, it is probable that the phenomenon we have identified here is more abundant, and the examples we detected reflect only a small portion of the actual shared splicing programs in cancer.

Our results suggest that several common splicing factors are responsible for the regulation of the pattern of splicing changes in different cancer types. Our data support a prominent regulatory role for *RBFOX2* and *QKI* as well as *PTBP1, MBNL1/2* and *CELF2* in at least three different cancer types (QKI and *RBFOX2* results are summarized in Table 4). These results extend recent studies establishing the role of *RBFOX2, QKI* and *PTBP1* in cancer (7,12,16,62). However, we did not detect all the splicing factors that were shown to influence cancer-regulated splicing, due either to the fact that they have degenerate motifs that were missed in our analysis, or do not regulate a large number of highly expressed splicing events. Interestingly, some of the splicing factors we identified were previously shown to have common targets (9,46,82,83). Moreover, *RBFOX2* was suggested to alter the splicing of the genes encoding QKI and *PTBP1* (74). Taken together, it is likely that these splicing factors work together on a common program, and also regulate one another as part of this program. Since *MBNL, QKI, RBFOX* and *PTBP* motifs were recently found to be enriched in introns flanking cassette exons that are associated with ES-cell differential splicing (84), and *QKI, PTBP1, MBNL1, CELF* and *RBFOX2* were shown to regulate differentiation in several different cell types (82,85–89), it may be postulated that altered expression of these proteins promote dedifferentiation and proliferation in differentiated cells as was shown for *MBNL* proteins (84).

**Table 4.** Summary of QKI and RBFOX2 results shown in this study

Tissue	QKI motif	QKI expression change	QKI KD correlation (+ positive, - negative)	RBFOX motif	RBFOX2 expression change	RBFOX2 KD correlation (+ positive, - negative)
BRCA	**+**	Downregulation	**+**	**+**	Downregulation	**+**
PRAD	**+**	Downregulation	**+**	**+**	Downregulation	**+**
LUAD	**+**	Downregulation	**+**	**+**		**+**
KIRC	**+**	Upregulation	**-**	**+**		
COAD	**+**	Downregulation	**+**	**+**	Downregulation	
LIHC			**+**			
HNSC			**-**	**+**		
THCA			**+**			

Cancer types are defined in Table 1.

Our study provides a global comparison of splicing pattern alteration and regulation in eight solid cancer types. Using TCGA data, we generated a basic catalog of splicing events associated with cancer in primary tumors in an unprecedented number of cancer types and in matched tumor-normal samples. Identifying cancer-regulated splicing events complements global expression profiling, and describes another layer of regulation that should be explored further. Our results can provide the basis for such follow-up studies designed for identification of splicing-based diagnostic/prognostic biomarkers and therapeutic targets for either one or several cancer types. Some of the common cancer-regulated alternative splicing events we identified in genes such as *FN1, FBLN2, AP2B1* and *TCF20* are most likely oncogenic drivers. Thus, modulation of their splicing by splice-site competing antisense oligonucleotides might be developed as a cancer therapy. Moreover, since common regulation was observed in several of the examined tumor types, deeper examination of the network effect of these splicing factors may help in obtaining a clearer understanding of cancer-altered splicing programs and splicing regulation in cancer.

## SUPPLEMENTARY DATA

Supplementary Data are available at NAR online.

SUPPLEMENTARY DATA

## Ethics statement

Human tumor samples from the tissue bank of the department of pathology of Hadassah Medical Center were obtained under the Institutional Helsinki committee approval no. 0574-10-HMO.
