# Supplementary material for: Identification of recurrent regulated alternative splicing events across human solid tumors
Source: Nucleic Acids Res. 2015 Apr 23;43(10):5130–44. doi: 10.1093/nar/gkv210 (PMC4446417; doi:10.1093/nar/gkv210)
Supplement: SUPPLEMENTARY DATA [file supp_43_10_5130__index.html]

Identification of recurrent regulated alternative splicing events across human solid tumors — SUPPLEMENTARY DATA 

# Identification of recurrent regulated alternative splicing events across human solid tumors

## SUPPLEMENTARY DATA

**Files in this Data Supplement:**

- SUPPLEMENTARY DATA
- SUPPLEMENTARY DATA
