# Supplementary material for: Identification of recurrent regulated alternative splicing events across human solid tumors
Source: Nucleic Acids Res. 2015 Apr 23;43(10):5130–44. doi: 10.1093/nar/gkv210 (PMC4446417; doi:10.1093/nar/gkv210)
Supplement: SUPPLEMENTARY DATA [file supp_gkv210_nar-03248-a-2014-File008.docx]

**Supplemental Material for**

**Recurrent regulated alternative splicing across human solid tumors**

Miri Gotthold-Danan^1^, Regina Golan-Gerstl^2^, Eli Eisenberg^3^, Keren Meir^4^, Rotem Karni^2*^ and Erez Y. Levanon^1*^

**Contents**

[Figure S1 2](#_Toc408511376)

[Figure S2 6](#_Toc408511377)

[Figure S3 7](#_Toc408511378)

[Figure S4. 10](#_Toc408511379)

[Figure S5 11](#_Toc408511380)

[Table S1 12](#_Toc408511382)

[Table S3 13](#_Toc408511383)

[Table S4 17](#_Toc408511384)

[Table S6 18](#_Toc408511385)

[Table S7 19](#_Toc408511386)

[Table S8 21](#_Toc408511387)

[Table S9 24](#_Toc408511388)

[Table S10 25](#_Toc408511389)

[Table S11 26](#_Toc408511390)

[Supplementary Methods 27](#_Toc408511391)

Table S2 and S5 are available in separate Excel files

BRCA


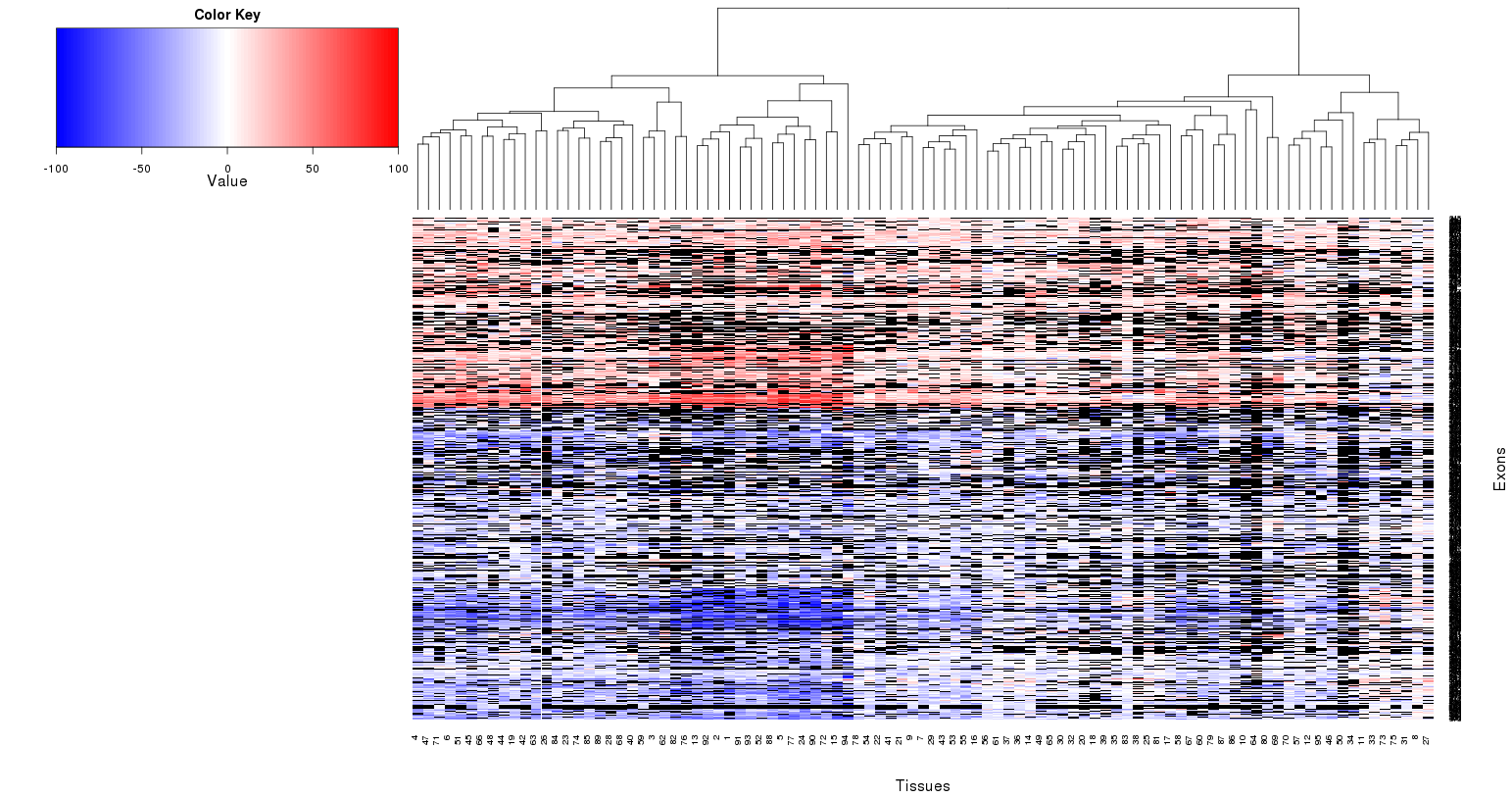


KIRC


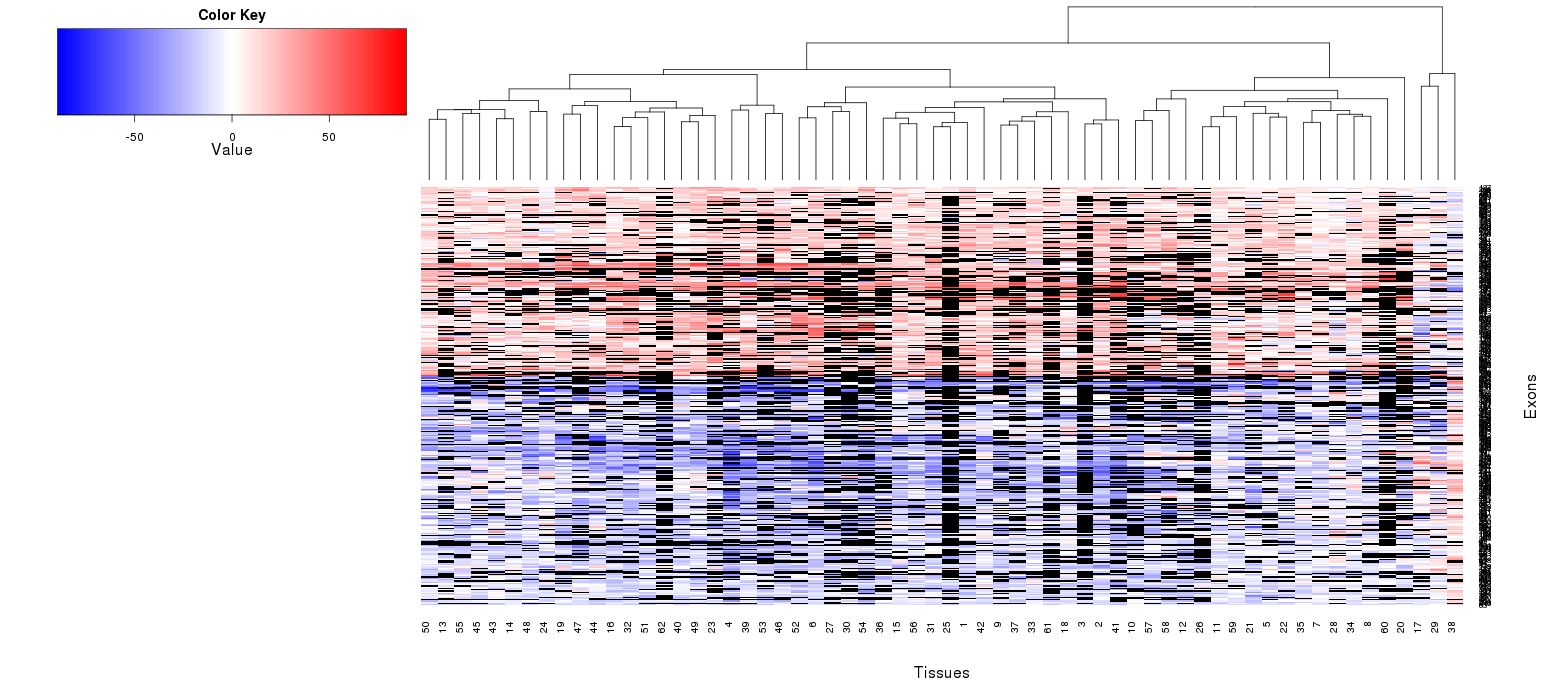


HNSC


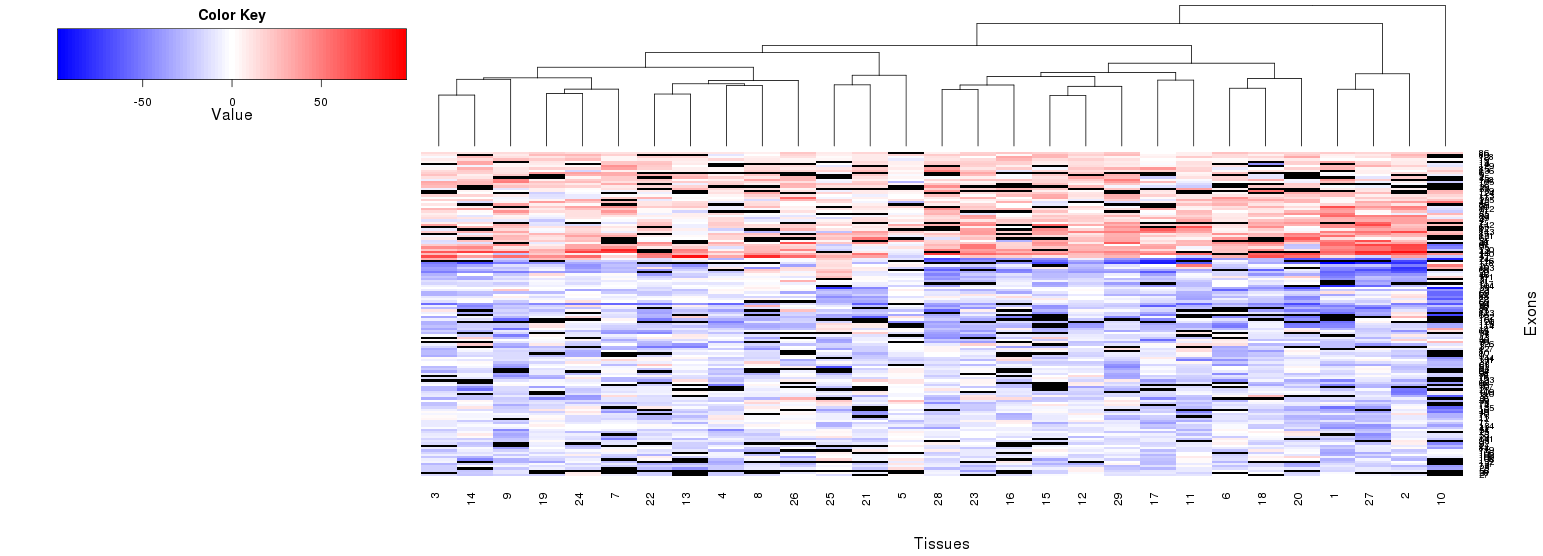


LUAD


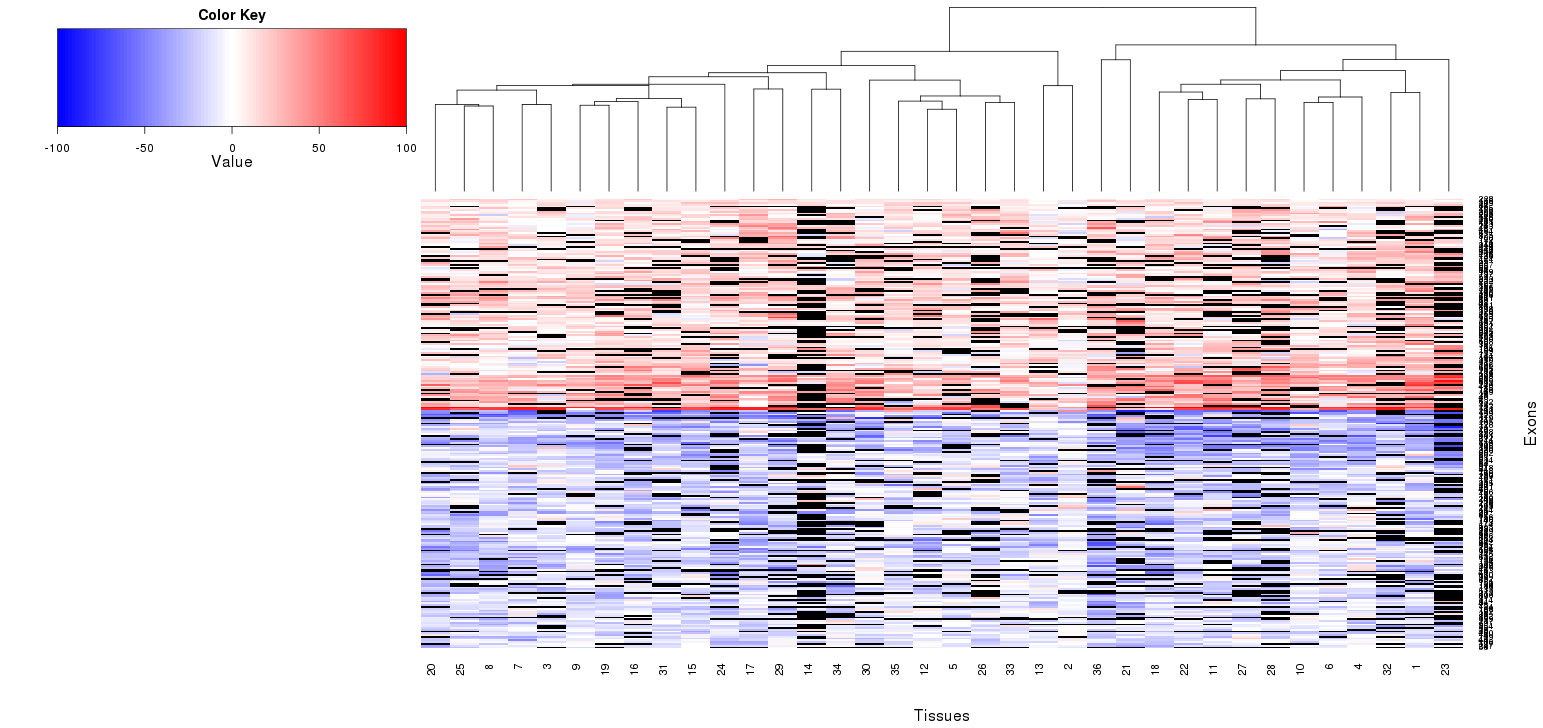


LIHC


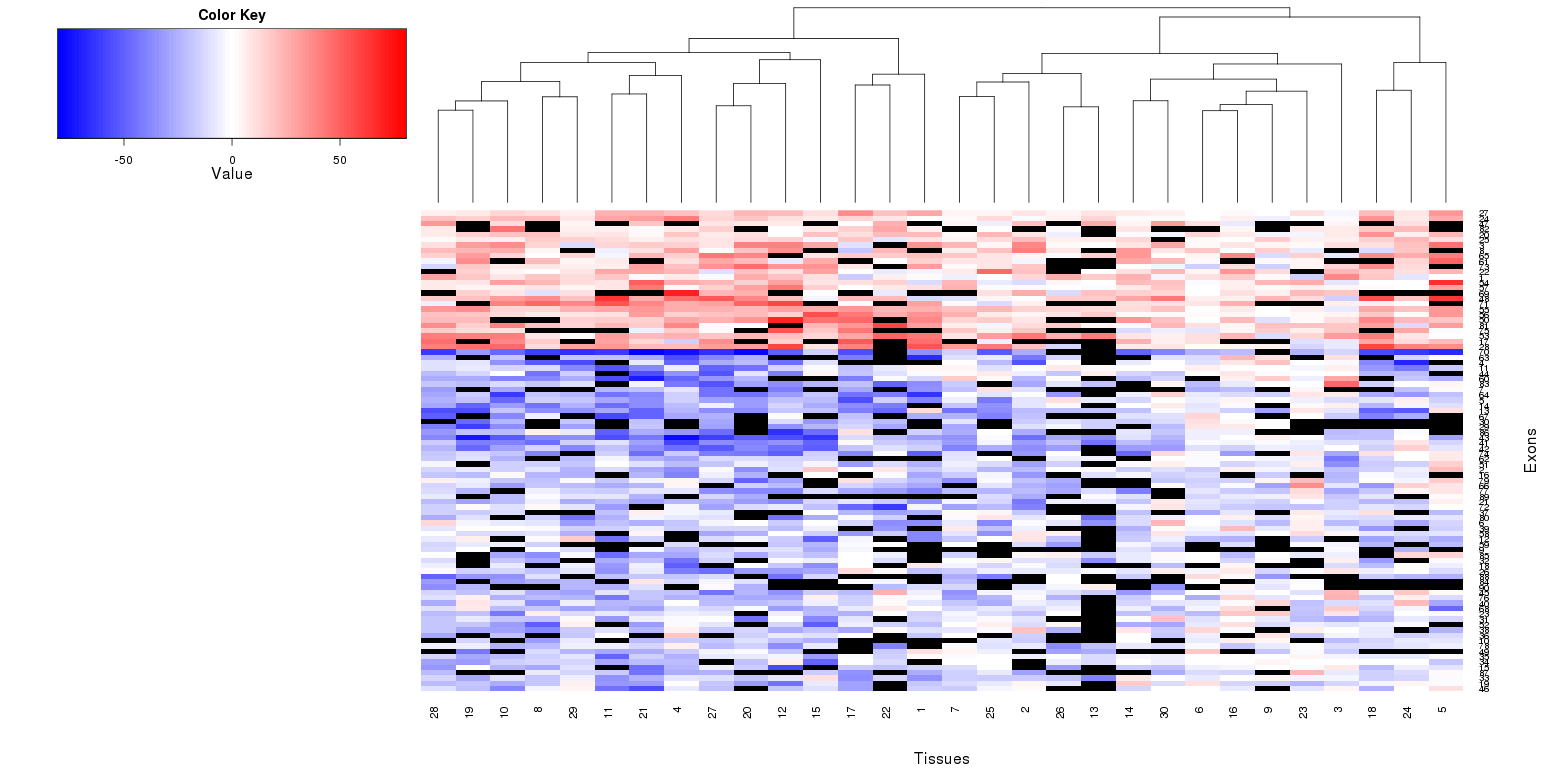


THCA


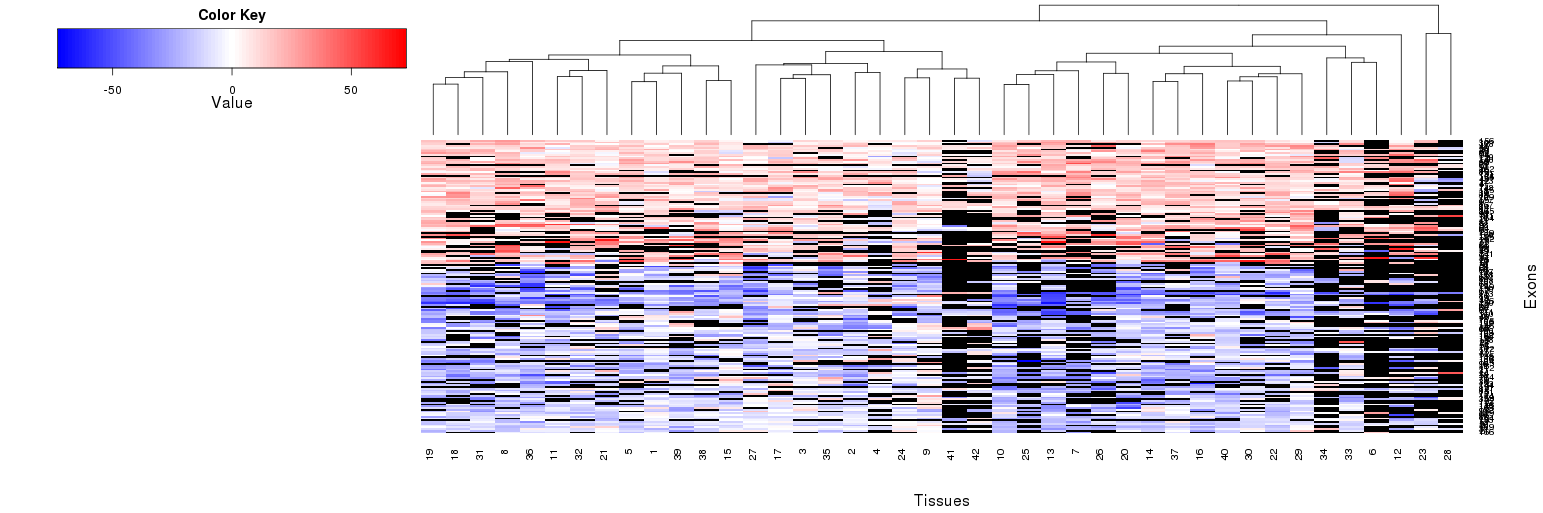


PRAD


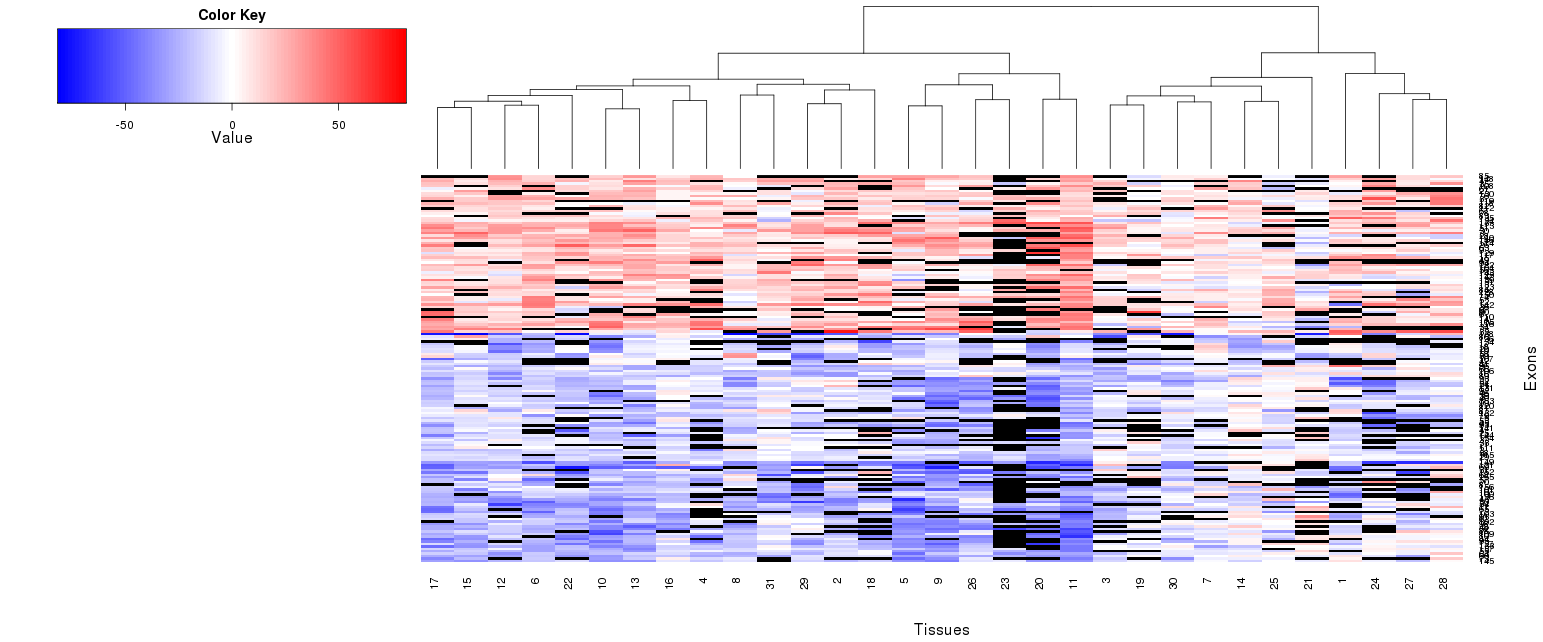


COAD


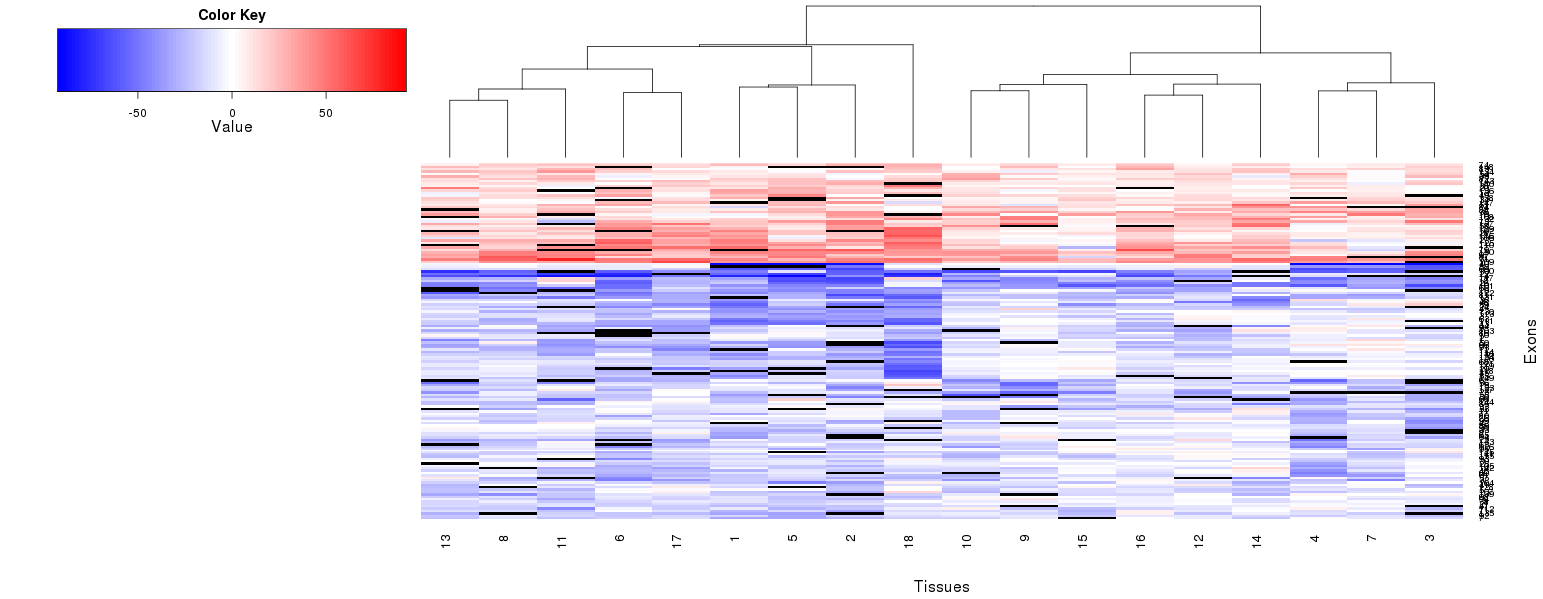


Figure S1. Heatmap of mean percent spliced in (PSI) shift (tumor – normal) for all differentially alternative splicing events in each cancer type. (Showing only splicing events supported by at least 20 and 16 samples in BRCA and KIRC, respectively)


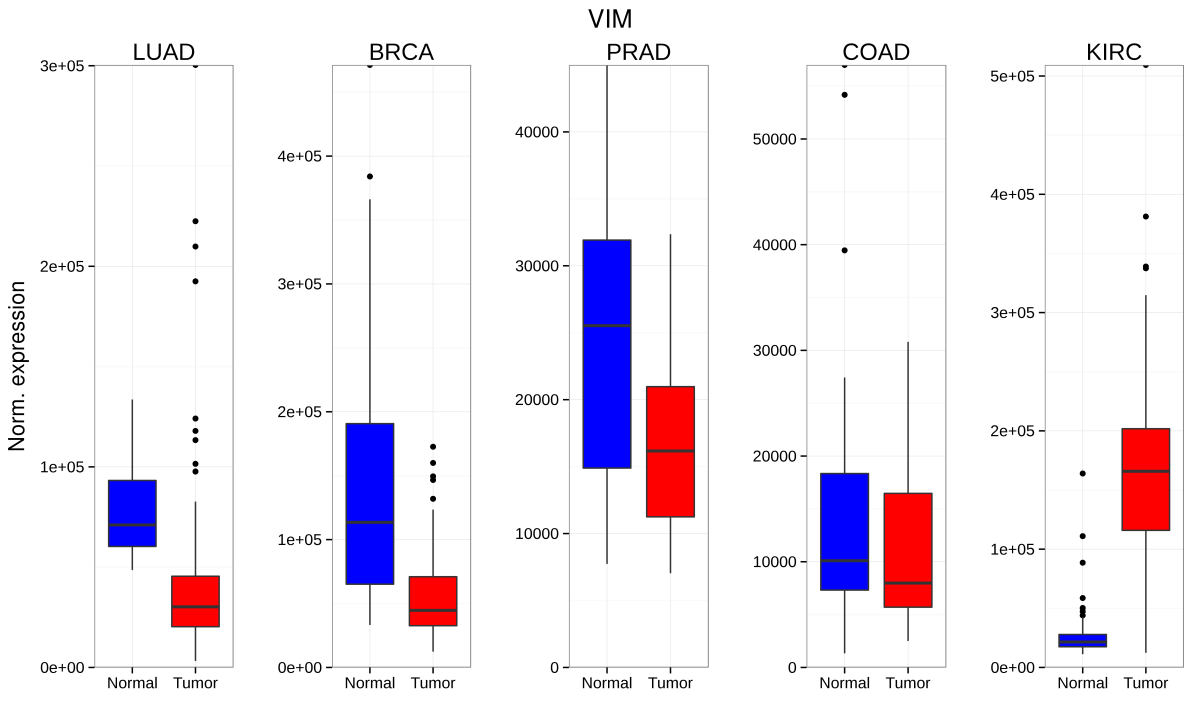


Figure S2. Boxplot representation of normalized expression level of normal and tumor samples for vimentin gene (VIM). Only cancer types with significant expression change between normal and tumor samples are shown (FDR<0.05).


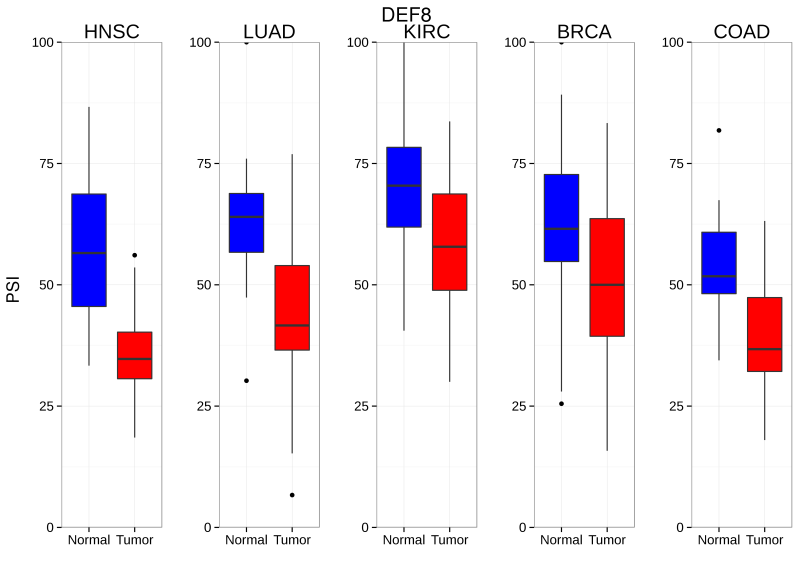

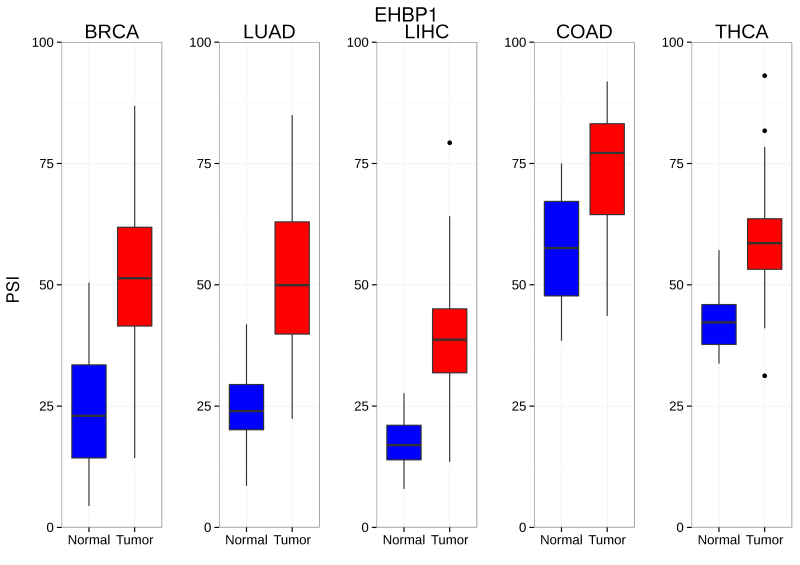

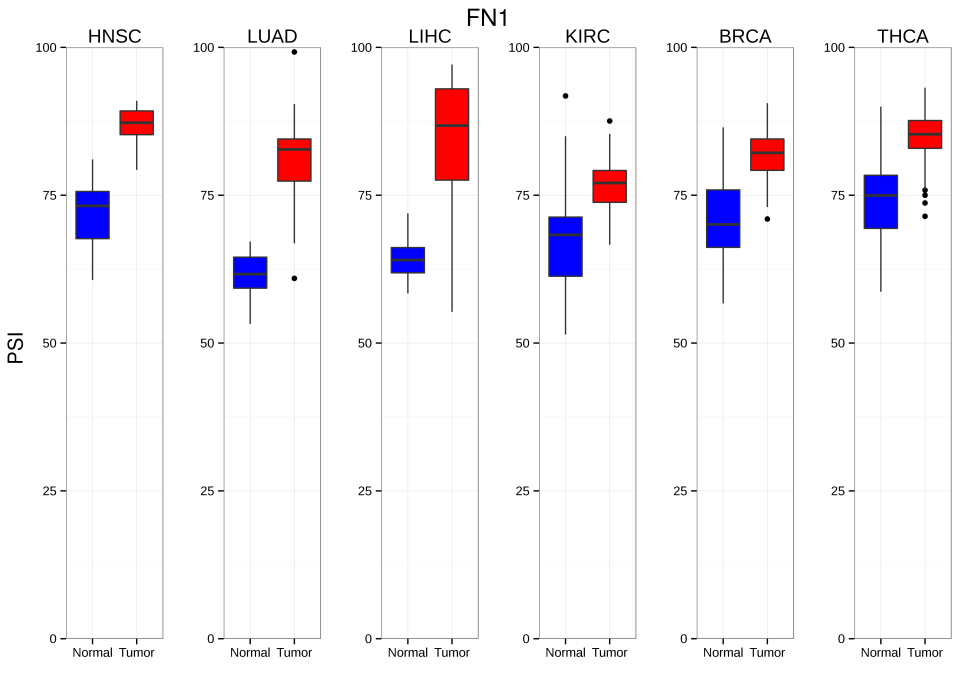

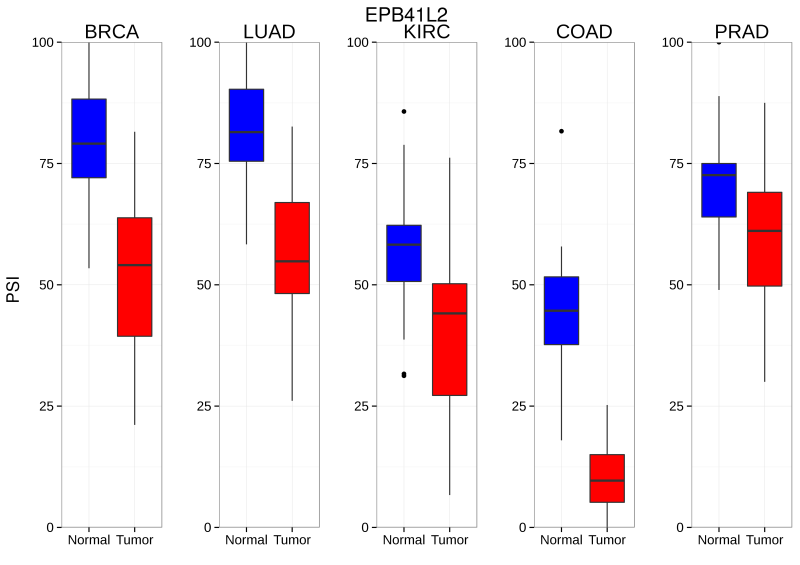

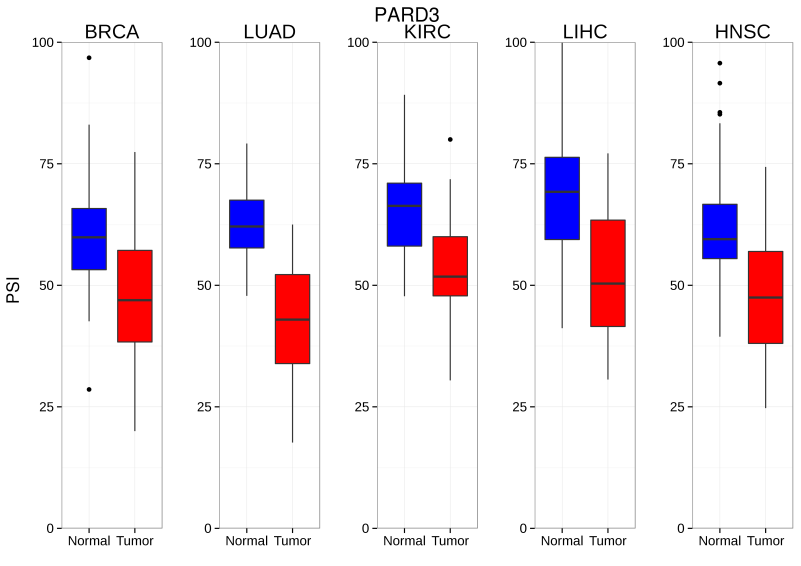

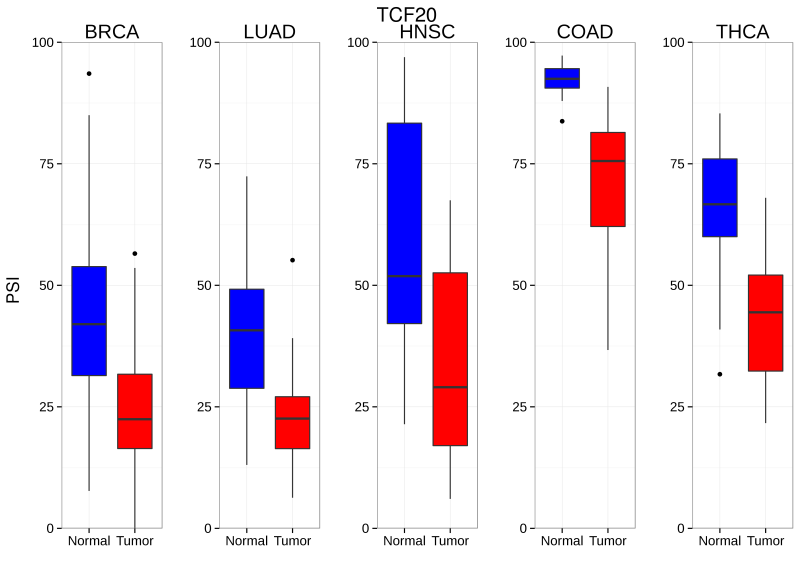

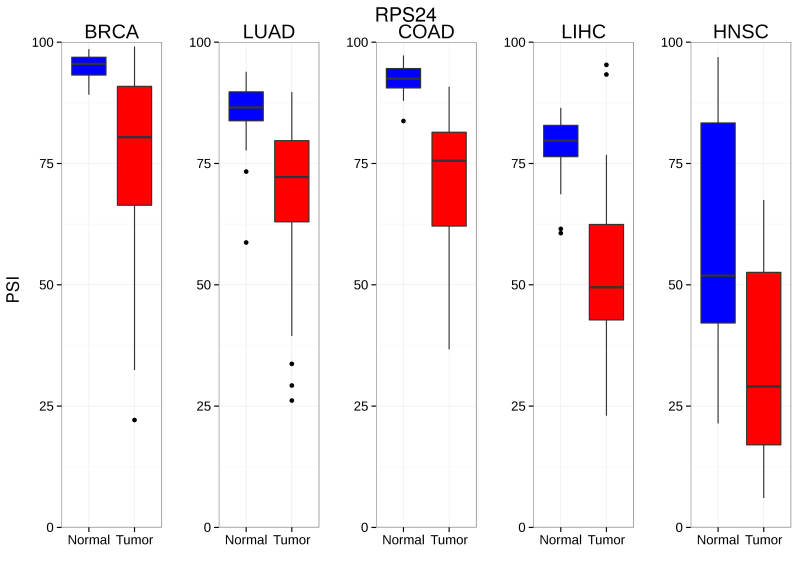

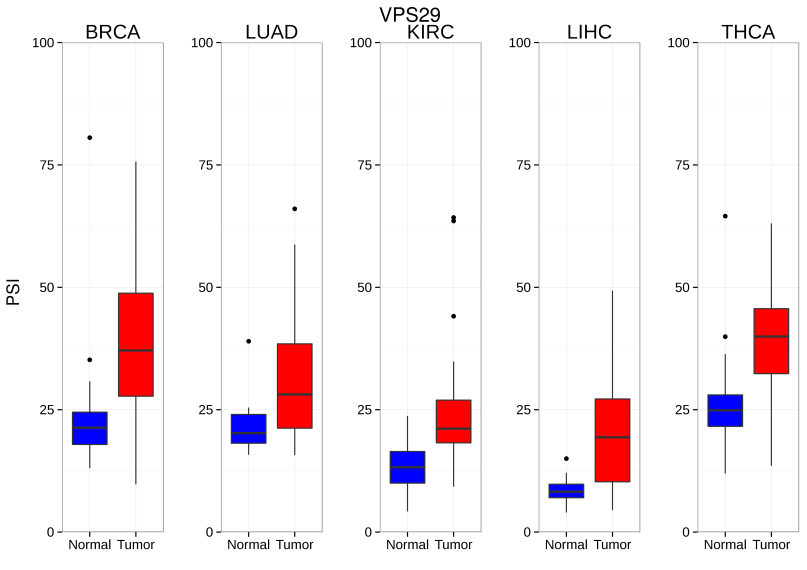


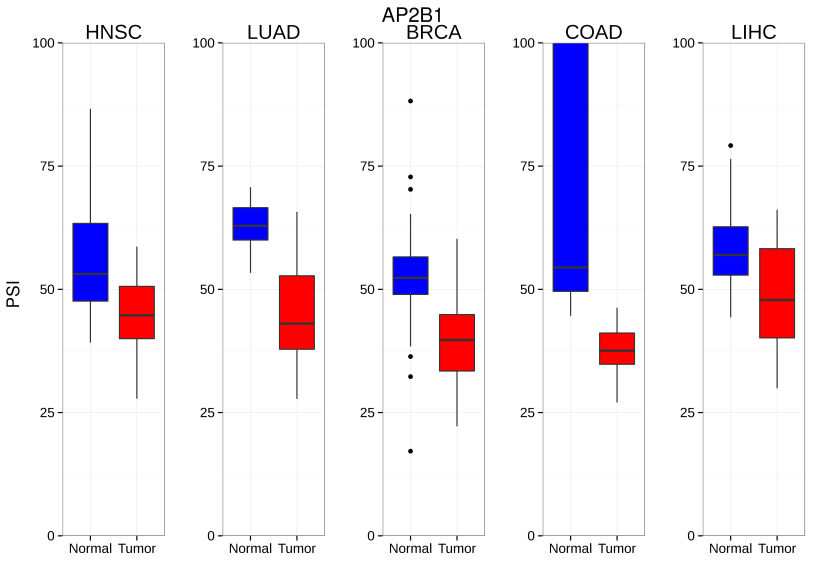


Figure S3. Boxplot representation of PSI level of normal and tumor samples for all the splicing events that altered in the same direction in at least 5 cancer types.


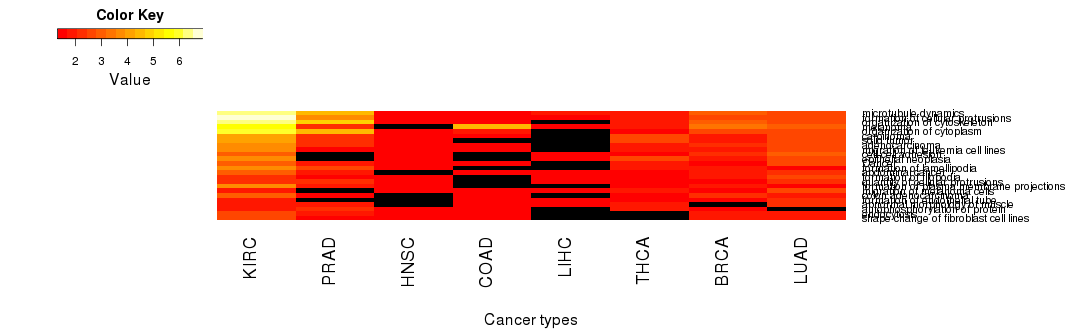

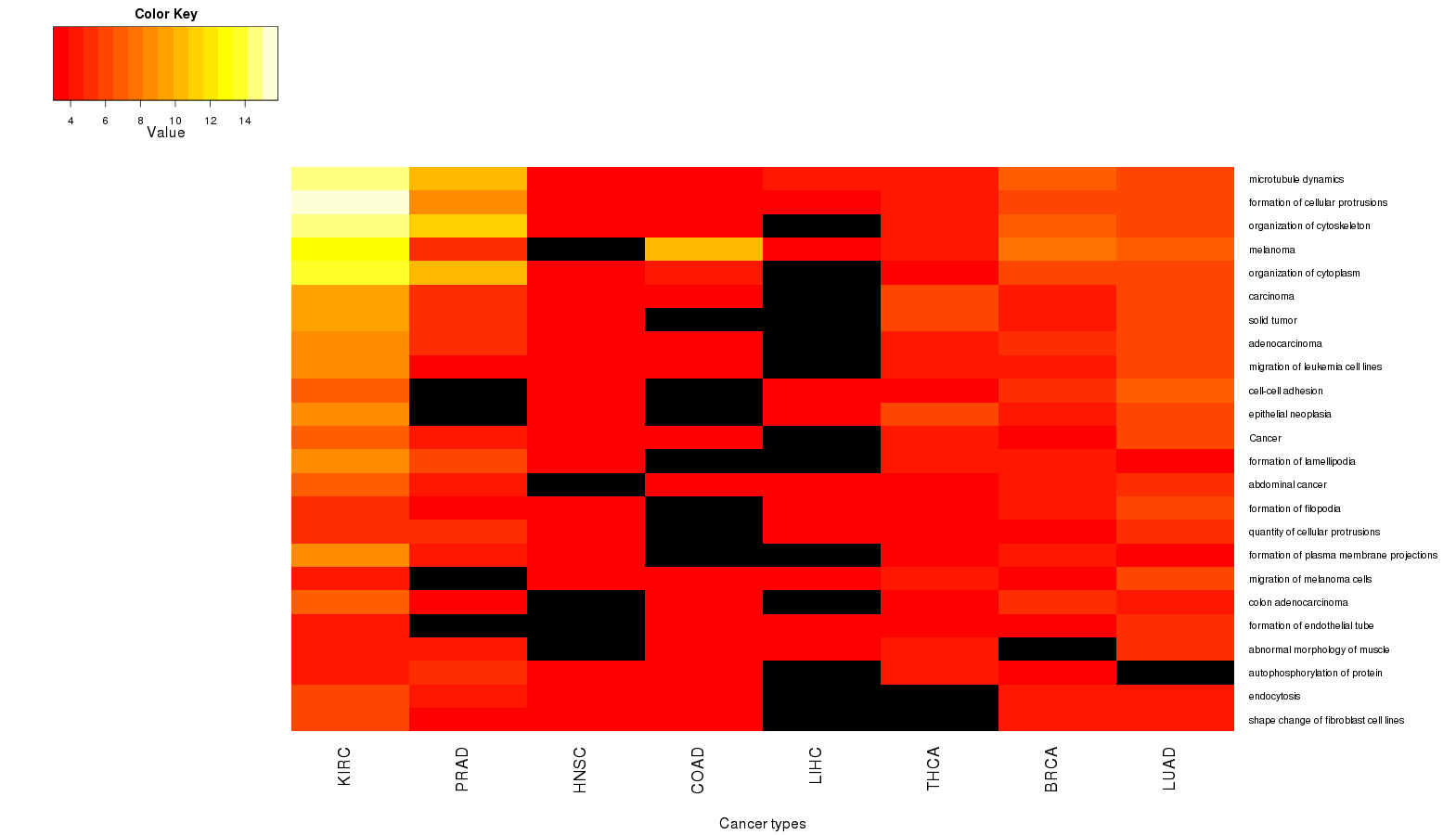


Figure S4. Common splicing program in solid cancers. Heatmap of –log(FDR) of the functional categories enriched (FDR<0.05) in at least 5 different cancer types.


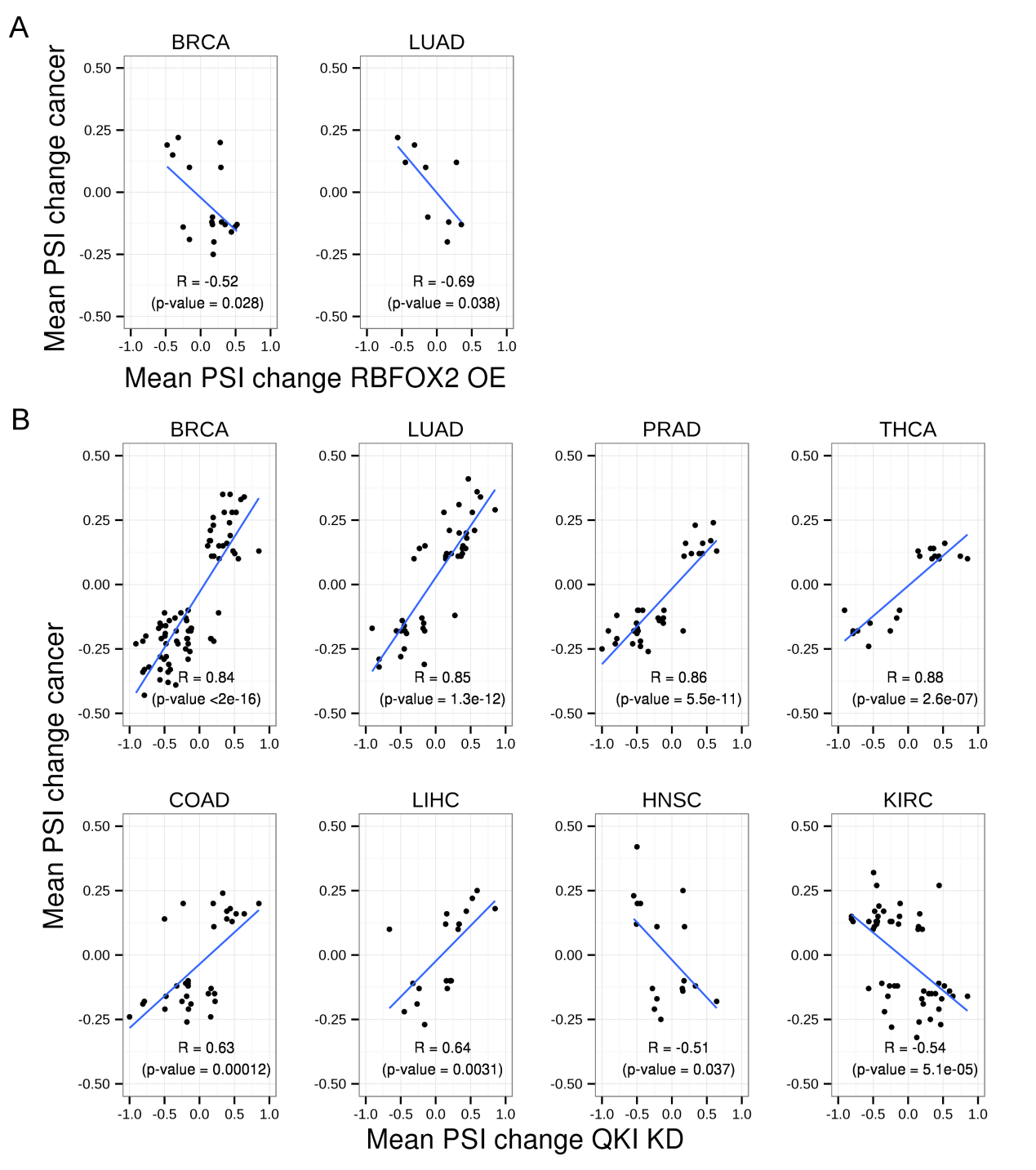


Figure S5. Correlation between RBFOX2 ectopic expression and QKI knockdown experiments and cancer splicing pattern changes. Scatter plot showing mean differences in PSIs of each cancer type (tumor versus normal samples) and mean differences in PSIs of (A) QKI and (B) RBFOX2;  (1, 2) experiment versus control treatments.

Table S1. **Summary of the cancer analysis results**

Global summary of the number of RNA-seq samples analyzed in this study and the cancer-associated splicing events

| **Dataset** | **# tumor samples** | **# normal samples** | **# matched samples** | **# highly expressed splicing events^A^** | **# differentially expressed splicing events^B^** | **# coherent change^C^** | **# high PSI -expression correlation^D^** | **% exons found in UCSC genes** | **% exons reserve reading frame^E^** |
| --- | --- | --- | --- | --- | --- | --- | --- | --- | --- |
| BRCA | 96 | 97 | 95 | 30,364 | 591 | 93 | 33 | 79.19% | 69.60% |
| KIRC | 66 | 65 | 62 | 15,648 | 337 | 61 | 10 | 81.60% | 75.67% |
| LIHC | 30 | 32 | 30 | 7,359 | 90 | 37 | 4 | 93.51% | 74.67% |
| LUAD | 291 | 43 | 36 | 20,225 | 240 | 116 | 0 | 89.58% | 75.65% |
| COAD | 144 | 20 | 18 | 8,846 | 140 | 115 | 2 | 89.28% | 76.12% |
| PRAD | 37 | 35 | 31 | 11,191 | 151 | 55 | 0 | 83.44% | 85.21% |
| HNSC | 33 | 30 | 29 | 12,095 | 146 | 61 | 2 | 87.67% | 68.57% |
| THCA | 48 | 48 | 42 | 14,268 | 157 | 58 | 1 | 82.80% | 71.72% |

A - Cassette exons in which all the junctions supporting the event were supported by at least 10 reads in at least 15 matched samples

B - Cassette exons in which there was a significant change in splicing pattern between normal and tumor samples

(|△PSI|>=10% and paired Wilcoxon test FDR<=0.05)

C - Cassette exons that the change (|△PSI|>10% ) in all examined matched samples was in one direction (inclusion/exclusion)

D- Pearson correlation between PSI and normalized expression (DE-seq) > 0.5

E – Percentage of exons divided by 3 out of total number of exons found in the UCSC/ENSMBL genes.

Table S3. **Summary of previously validated splicing pattern change in cancer**

| **Gene** | **Exon** | **Pattern change** | **Cancer type** | **Cancer type in this study** | **Pattern change in this study** | **Type of validation** | **Ref.** |
| --- | --- | --- | --- | --- | --- | --- | --- |
| RAC1 | 3b | Inclusion | COAD | LUAD, KIRC | Inclusion (LUAD), Exclusion (KIRC) | Thorough analysis | (3, 4) |
| MACF1 | 8 | Inclusion | NSCLC | LUAD,KIRC, COAD, LIHC | Inclusion (LUAD),  Exclusion (KIRC, COAD, LIHC) | RT-PCR | (5) |
| NUMB | 9 | Inclusion | LUAD, NSCLC | LUAD, BRCA, PRAD, LIHC, KIRC | Inclusion (LUAD, BRCA, PRAD, LIHC), Exclusion (KIRC) | RT-PCR, protein | (1, 5) |
| VEGFA | 6 | Exclusion | Enhanced proliferation | BRCA, LUAD, LIHC | Exclusion | Thorough analysis | (6) |
| APP | 8 | Exclusion | NSCLC | BRCA, PRAD, KIRC | Exclusion (BRCA, PRAD), Inclusion (KIRC) | RT-PCR | (5) |
| APLP2 | 7 | Exclusion | BRCA  (cell line) | LUAD, LIHC, KIRC | Exclusion | RT-PCR | (7) |
| MYL6 | 6 | Exclusion | BRCA  (cell line) | BRCA, HNSC | Exclusion (BRCA) , Inclusion (HNSC) | RT-PCR | (7) |
| ZNF207 | 9 | Exclusion | BRCA  (cell lines) | BRCA, PRAD | Exclusion | RT-PCR | (7) |
| CTTN | 11 | Inclusion | COAD | COAD | Inclusion | RT-PCR | (8) |
| BIN1 | 12a | Inclusion | PRAD, neuroblastoma, melanoma, NSCLC | LIHC, LUAD, KIRC, THCA | Inclusion | Thorough analysis | (1, 9–11) |
| CEACAM1 | 7 | Exclusion,  Inclusion | NSCLC, BRCA | LIHC, LUAD, KIRC | Inclusion (LIHC), exclusion (KIRC, LUAD) | qRT-PCR, RT-PCR | (9, 12, 13) |
| PTK6 | 2 | Inclusion | PRAD | COAD | Exclusion | Thorough analysis | (14) |
| FGFR1 | 3 (alpha exon) | Exclusion | Glioblastoma multiforme, Prostate adenocarcinoma | KIRC | Exclusion | PCR | (15, 16) |
| INSR | 11 | Exclusion | BRCA, LUAD, COAD | LIHC, BRCA, KIRC | Exclusion | RT-PCR | (17) |
| ARMC10 (SVH) | 2 | Exclusion | LIHC | HNSC | Inclusion | Thorough analysis | (18) |
| SYK | 7 | Exclusion | BRCA | KIRC, HNSC, THCA | Inclusion | Thorough analysis | (19) |
| TNC | 10-16 | Inclusion | BRCA, LIHC, COAD, oral squamous cell carcinoma, LUAD, PRAD, brain tumors | BRCA, LUAD, KIRC, HNSC | Inclusion | Thorough analysis | (20–23) |
| FN1 | EDA (270 nt) | Inclusion | LIHC, COAD, GBM | LIHC, BRCA, HNSC | Inclusion | Thorough analysis | (24, 25) |
| FN1 | EDB (267 nt) | Inclusion | LIHC, COAD | LIHC, BRCA, KIRC, HNSC, LUAD, THCA | Inclusion | Thorough analysis | (24, 25) |
| FN1 | 273 nt | Inclusion | NSCLC | LIHC, LUAD, THCA | Inclusion | RT-PCR | (1) |
| KIAA1109 | 4 | High inclusion rate correlated with poor prognosis in patients with breast cancer | BRCA | BRCA, PRAD, HNSC,  KIRC | Inclusion (BRCA, PRAD), Exclusion (HNSC, KIRC) | qRT-PCR | (26) |
| EPB41 | 22 | High inclusion rate correlated with poor prognosis in patients with breast cancer | BRCA | BRCA, LUAD, PRAD, KIRC | Inclusion (BRCA, LUAD, PRAD), Exclusion (KIRC) | qRT-PCR | (26) |
| CLSTN1 | 11 | Low inclusion rate correlated with poor prognosis in patients with breast cancer | BRCA | BRCA, LUAD, PRAD, KIRC | Exclusion (BRCA, LUAD, PRAD), exclusion (KIRC) | qRT-PCR | (26) |
| NF1 | 23aa (63bp) | Exclusion | Brain tumors | BRCA | Exclusion | RT-PCR | (23, 27) |
| VCL | 19 | Exclusion | COAD, PRAD, bladder | PRAD, COAD | Exclusion | RT-PCR | (8, 28) |
| CALD1 | 6 | Exclusion | GBM, COAD | BRCA | Exclusion | RT-PCR | (23) |
| ATP2B4 | 21 | Exclusion | COAD | COAD | Exclusion | RT-PCR | (8) |
| GK | 20 | Inclusion | COAD | COAD | Inclusion | RT-PCR | (8) |
| COL6A3 | 6 (200aa) | Inclusion | COAD, PRAD, bladder | BRCA, LUAD | Inclusion | RT-PCR | (8, 28) |
| PI4KB | 4 | Exclusion (COAD, bladder)/ Inclusion (PRAD) | COAD, bladder, PRAD | KIRC | Exclusion | RT-PCR | (28) |
| FBLN2 | 9 | Exclusion, Inclusion | LUAD, nasopharyngeal cancer | BRCA, LUAD, COAD, HNSC, THCA | Exclusion | Thorough analysis | (29, 30) |
| TCF7L2 | 4 | Exclusion | LIHC | LUAD, KIRC | Inclusion (LUAD), Exclusion (KIRC) | Thorough analysis | (31) |
| ITGB4 | 35 | Exclusion | COAD | BRCA, LUAD, THCA | Exclusion | RT-PCR | (8) |
| ADD3 | 15 | Inclusion | NSCLC | LIHC, LUAD, BRCA, COAD, KIRC, THCA | Inclusion (LUAD, LIHC, BRCA, COAD, THCA), Exclusion (KIRC) | RT-PCR | (1) |
| EXOC1 | 11 | Inclusion | NSCLC | BRCA, LUAD, PRAD, KIRC, HNSC, COAD | Inclusion (BRCA, LUAD, PRAD, COAD), Exclusion (KIRC, HNSC) | RT-PCR | (1) |
| MYO18A | 40 | Exclusion | NSCLC | BRCA, LUAD, PRAD, KIRC | Inclusion (BRCA, LUAD, PRAD) Exclusion (KIRC) | RT-PCR | (1) |
| NFYA | 3 | Exclusion | NSCLC | BRCA, LUAD, KIRC | Exclusion (BRCA, LUAD), Inclusion (KIRC) | RT-PCR | (1) |
| CLSTN1 | 3 | Exclusion | NSCLC | BRCA, LUAD | Exclusion | RT-PCR | (1) |
| EHBP1 | 17 | Inclusion | NSCLC | LIHC, BRCA, LUAD, COAD, THCA | Inclusion | RT-PCR | (1) |
| ESYT2 | 5 | Inclusion | NSCLC | BRCA, LUAD, KIRC, COAD | Inclusion (BRCA, LUAD, COAD) Exclusion (KIRC) | RT-PCR | (1) |
| KIF13A | 38 | Inclusion | NSCLC | LIHC, BRCA, LUAD, PRAD, KIRC, COAD | Inclusion (BRCA, LUAD, COAD, PRAD, LIHC) Exclusion (KIRC) | RT-PCR |  |
| ITGB4 | 34b | Exclusion | Lung, Kidney, Colon, Uterus (The type of cancer was not specified) | BRCA, LUAD, THCA | Exclusion | RT-PCR | (32) |
| NGLY1 | 10 | Exclusion | Lung, uterus (The type of cancer was not specified) | BRCA | Exclusion | RT-PCR | (32) |

| **LUAD** | **COAD** | **BRCA** | **Splicing event** |
| --- | --- | --- | --- |
| 5/5 | 5/5 | 4/4 | EPB41L2  Decreased inclusion  tumor/normal |
| 4/5 | 5/5 | 4/4 | FBNL2  Decreased inclusion  tumor/normal |
| 5/5 | 5/5* | 2/4 | PARD3  Decreased inclusion  tumor/normal |
| 3/5 | 2/4* | 4/4 | TCF20  Decreased inclusion  tumor/normal |
| 5/5* | 4/5* | 4/4 | WDFY3  Increased inclusion  tumor/normal |
| 4/5 | 3/5 | 4/4 | AP2B1  Decreased inclusion  tumor/normal |
| 5/5 | 4/5 | 4/4* | MBNL1  Increased inclusion  tumor/normal |
| 5/5 | - | - | SCEL  Increased inclusion  tumor/normal |

Table S4. **Summary of RT-PCR validation results**

The number of matched samples with the expected change observed in the RT-PCR validation (quantitatively quantified) out of the number of matched samples examined is indicated. Splicing events that were examined in additional cancer type (other than the predicted) are indicated by an asterisk.

# Table S6. Enriched motifs in introns upstream of cancer-excluded exons

| **Sequence** | **Tissue** | **FDR** |
| --- | --- | --- |
| TTCTCT | PRAD | 1.23E-06 |
| CTCTCT | BRCA | 1.08E-04 |
| GTTCCT | BRCA | 1.08E-04 |
| CTCTTC | KIRC | 1.08E-04 |
| TCTCT | PRAD | 1.53E-04 |
| TGTT | PRAD | 3.12E-04 |
| TTCCT | BRCA | 4.55E-04 |
| TCTCTC | PRAD | 5.47E-04 |
| TCTCTC | BRCA | 5.79E-04 |
| TTTCTC | BRCA | 8.39E-04 |
| TCTCT | BRCA | 8.39E-04 |
| CTCTCT | PRAD | 9.54E-04 |
| TGCATG | HNSC | 1.20E-03 |
| TTTCTC | PRAD | 2.07E-03 |
| CTCTT | BRCA | 2.07E-03 |
| GGCAAT | HNSC | 3.35E-03 |
| CTTGCT | BRCA | 3.62E-03 |
| TTTTC | BRCA | 4.32E-03 |
| TGTTC | BRCA | 5.10E-03 |
| CTGTTT | PRAD | 5.38E-03 |
| TTGCT | KIRC | 5.88E-03 |
| TTCTCT | BRCA | 6.98E-03 |
| ATTTC | BRCA | 7.28E-03 |
| TCTCTT | BRCA | 8.96E-03 |
| TGATTA | PRAD | 8.96E-03 |
| CGTCGG | HNSC | 8.96E-03 |
| TCTCT | KIRC | 8.96E-03 |

| RBFOX1/2 motif or related sequence  (33) |
| --- |
| CELF motif or related sequence  (34, 35) |
| MBNL motif or related sequence  (36) |
| PTB motif or related sequence  (37–39) |
|  |
|  |
|  |
|  |

# **Table S7**. Enriched motifs in introns upstream of cancer-included exons

| RBFOX1/2 motif or related sequence  (33)  QKI motif or related sequence  (40) |
| --- |
| CELF motif or related sequence  (34, 35) |
| MBNL motif or related sequence  (36) |
|  |
|  |
|  |
|  |
|  |

| **Sequence** | **Tissue** | **FDR** |
| --- | --- | --- |
| TGCATG | BRCA | 2.34E-07 |
| GCATG | BRCA | 3.45E-06 |
| ACTAAC | LUAD | 5.04E-05 |
| TAACA | LUAD | 6.59E-05 |
| ACTAAC | BRCA | 1.05E-04 |
| TTAAC | COAD | 1.11E-04 |
| TGTTTT | LUAD | 2.17E-04 |
| ATTAAC | COAD | 5.64E-04 |
| TGTTT | LUAD | 5.64E-04 |
| TTGCT | COAD | 5.64E-04 |
| GCATGC | COAD | 1.44E-03 |
| TCAT | LUAD | 1.44E-03 |
| TGCATG | COAD | 1.47E-03 |
| TTAAC | LUAD | 1.47E-03 |
| TTGCTT | BRCA | 1.75E-03 |
| TAAC | LUAD | 1.75E-03 |
| TAACAC | LUAD | 1.75E-03 |
| TTGCT | LUAD | 1.75E-03 |
| TGCAT | COAD | 1.84E-03 |
| CCCCCC | BRCA | 2.21E-03 |
| GCATGC | BRCA | 2.22E-03 |
| TGCTTC | BRCA | 2.22E-03 |
| CTAAC | LUAD | 2.22E-03 |
| TGCAT | LUAD | 2.22E-03 |
| TGCATG | LUAD | 2.86E-03 |
| ACCGGT | THCA | 2.86E-03 |
| TTGCTT | LIHC | 3.16E-03 |
| GTTTT | LUAD | 3.18E-03 |
| TTGC | COAD | 3.18E-03 |
| GTGCAT | COAD | 3.25E-03 |
| TTAACC | COAD | 3.27E-03 |
| AACT | LIHC | 3.27E-03 |
| TGCATG | PRAD | 3.44E-03 |
| TAACAT | LUAD | 3.55E-03 |
| CTAAC | BRCA | 4.48E-03 |
| GCATGG | BRCA | 5.38E-03 |
| CATTCC | KIRC | 5.38E-03 |
| CTAACC | BRCA | 5.44E-03 |
| TGCAT | BRCA | 5.90E-03 |
| CGCCGA | BRCA | 6.00E-03 |
| TAACA | COAD | 6.18E-03 |
| ATCAT | LUAD | 7.54E-03 |
| TGTT | LUAD | 7.60E-03 |
| CTAACC | LUAD | 7.63E-03 |
| TTGGTT | COAD | 8.46E-03 |
| TAACC | LUAD | 8.46E-03 |
| TGCTT | COAD | 8.46E-03 |
| TAAC | COAD | 8.71E-03 |
| AATCAT | LUAD | 9.00E-03 |

# **Table S8**. Enriched motifs in introns downstream of cancer- excluded exon

| RBFOX1/2 motif or related sequence (33) |
| --- |
| QKI motif or related sequence  (40) |
| MBNL motif or related sequence  (36) |

| **Sequence** | **Tissue** | **FDR** |
| --- | --- | --- |
| TGCATG | BRCA | 2.30E-26 |
| TGCAT | BRCA | 3.41E-15 |
| ACTAAC | BRCA | 7.82E-13 |
| GCATGC | BRCA | 5.49E-12 |
| GCATG | BRCA | 5.50E-10 |
| TGCATG | PRAD | 1.82E-09 |
| CACTAA | BRCA | 4.55E-09 |
| CTAAC | BRCA | 4.30E-08 |
| CTGCAT | BRCA | 6.54E-08 |
| ACTAA | BRCA | 7.08E-08 |
| TGCAT | PRAD | 1.63E-07 |
| TAAC | BRCA | 4.20E-07 |
| CTAAC | PRAD | 4.58E-07 |
| CTGCAT | PRAD | 8.68E-07 |
| TAACAC | BRCA | 1.16E-06 |
| ACTAAC | LUAD | 1.59E-06 |
| TGCATG | COAD | 6.18E-06 |
| CATGC | BRCA | 6.18E-06 |
| CCGCT | BRCA | 6.18E-06 |
| CTTGC | BRCA | 1.12E-05 |
| CCGCTT | BRCA | 2.27E-05 |
| TGCATG | KIRC | 2.27E-05 |
| GCTT | BRCA | 2.27E-05 |
| TTGC | BRCA | 2.27E-05 |
| CTCCCC | BRCA | 3.77E-05 |
| GCATG | PRAD | 4.53E-05 |
| GCATGT | BRCA | 1.45E-04 |
| CGCTT | BRCA | 1.45E-04 |
| CCGC | BRCA | 1.45E-04 |
| ACTAAC | PRAD | 1.59E-04 |
| GTGCAT | BRCA | 1.69E-04 |
| TATCCG | THCA | 2.44E-04 |
| CTAACC | PRAD | 2.51E-04 |
| CATGCA | BRCA | 2.62E-04 |
| TAAC | PRAD | 3.05E-04 |
| GCATGC | PRAD | 4.93E-04 |
| ATGCAT | BRCA | 5.19E-04 |
| CATGCT | BRCA | 6.06E-04 |
| TGCTT | BRCA | 6.09E-04 |
| GCAT | BRCA | 6.16E-04 |
| CACTA | BRCA | 6.67E-04 |
| GCATGC | KIRC | 7.57E-04 |
| GCTTT | BRCA | 7.59E-04 |
| TCAAT | THCA | 7.66E-04 |
| CTAACC | BRCA | 8.42E-04 |
| TTGCT | BRCA | 9.66E-04 |
| TGTGT | BRCA | 1.07E-03 |
| TTGCAT | BRCA | 1.14E-03 |
| TGCAT | COAD | 1.14E-03 |
| TACTT | THCA | 1.15E-03 |
| CCATCG | BRCA | 1.29E-03 |
| GTCCGC | BRCA | 1.44E-03 |
| TGTCCT | KIRC | 1.44E-03 |
| ATGCAT | PRAD | 1.55E-03 |
| ACTAAT | BRCA | 1.64E-03 |
| TGCCCC | BRCA | 1.64E-03 |
| GCCAAT | BRCA | 1.83E-03 |
| TTGCA | BRCA | 2.07E-03 |
| TTTGC | BRCA | 2.10E-03 |
| CCCGC | BRCA | 2.13E-03 |
| TCTAAC | PRAD | 2.29E-03 |
| CCTTGC | BRCA | 2.73E-03 |
| ACTAAC | COAD | 2.80E-03 |
| CGCTTT | BRCA | 2.93E-03 |
| CTCCTT | BRCA | 2.93E-03 |
| ACTAAC | KIRC | 2.93E-03 |
| GCATGG | BRCA | 3.16E-03 |
| GTGCAT | PRAD | 3.16E-03 |
| TGCAT | KIRC | 3.16E-03 |
| TGTCT | KIRC | 3.18E-03 |
| TTGCTT | BRCA | 3.51E-03 |
| ATCTAA | THCA | 3.68E-03 |
| GGGGCT | LIHC | 3.73E-03 |
| CGCTTC | BRCA | 3.78E-03 |
| ATGCA | BRCA | 3.78E-03 |
| TCCGCT | THCA | 3.96E-03 |
| TACTAA | BRCA | 4.13E-03 |
| CCATCA | BRCA | 4.14E-03 |
| CATGGC | BRCA | 4.42E-03 |
| TTTGCA | BRCA | 4.74E-03 |
| CATG | BRCA | 5.48E-03 |
| TTGCA | PRAD | 5.58E-03 |
| CTAAC | COAD | 5.58E-03 |
| TCCTTG | BRCA | 5.78E-03 |
| GTTTGC | BRCA | 5.85E-03 |
| TGTGC | BRCA | 6.27E-03 |
| GTCGAG | BRCA | 6.62E-03 |
| GCTTTT | BRCA | 6.79E-03 |
| GTTTG | BRCA | 6.79E-03 |
| CTAACA | BRCA | 6.81E-03 |
| ACTAA | PRAD | 6.83E-03 |
| TTACTT | THCA | 6.98E-03 |
| TAACA | BRCA | 7.23E-03 |
| CTTGCT | BRCA | 7.45E-03 |
| GCTTGC | BRCA | 7.59E-03 |
| TTACT | THCA | 7.77E-03 |
| AAATC | THCA | 7.92E-03 |
| CTAACG | PRAD | 8.09E-03 |
| CCCGCT | BRCA | 8.14E-03 |
| TTTGTG | BRCA | 8.92E-03 |
| ATGCAC | BRCA | 8.96E-03 |
| TGCCTC | LIHC | 9.14E-03 |
| AGCACG | COAD | 9.14E-03 |
| CCCGCT | COAD | 9.14E-03 |
| CACTAA | LUAD | 9.48E-03 |
| ATACTA | KIRC | 9.75E-03 |
| TGCA | PRAD | 9.92E-03 |

|  |
| --- |
|  |
|  |
|  |

# **Table S9**. Enriched motifs in introns downstream of cancer-included exons

| **Sequence** | **Tissue** | **FDR** |
| --- | --- | --- |
| GGGGGG | BRCA | 1.20E-03 |
| CCGCT | BRCA | 1.20E-03 |
| AATCA | PRAD | 1.20E-03 |
| TGCATG | KIRC | 2.50E-03 |
| TCAATG | PRAD | 4.26E-03 |
| TAAGG | LIHC | 4.26E-03 |
| AATCA | LUAD | 4.52E-03 |
| TACGAG | BRCA | 5.52E-03 |
| ATGACG | BRCA | 7.65E-03 |
| CTCTTC | BRCA | 7.65E-03 |
| TTACGA | BRCA | 7.65E-03 |
| GTCGCG | COAD | 7.65E-03 |
| CAGACA | BRCA | 7.73E-03 |
| AACCAC | LIHC | 8.16E-03 |
| ACGAGT | LUAD | 8.16E-03 |
| AGCCGC | LUAD | 8.16E-03 |

| RBFOX1/2 motif or related sequence  (33) |
| --- |
| MBNL motif or related sequence  (36) |
| PTB motif or related sequence  (37–39) |
|  |
|  |
|  |
|  |

Table S10. **Differential expression of splicing factors that their motif was significantly enriched in the introns flanking cancer-associated exons.**

| **Splicing factor** | **Dataset** | **Fold change** | **Enriched motif** | **Position and PSI change in cancer** |
| --- | --- | --- | --- | --- |
| RBFOX2 | COAD | 0.71 (p-value = 0.01) | + | Downstream to exons with higher exclusion  Upstream to exons with higher inclusion |
| RBFOX2 | BRCA | 0.65 (p-value = 0.007) | + | Downstream to exons with higher exclusion  Upstream to exons with higher inclusion |
| RBFOX2 | PRAD | 0.74 (p-value = 0.04) | + | Downstream to exons with higher exclusion  Upstream to exons with higher inclusion |
| QKI | BRCA | 0.46 (p-value = 6.29e-06) | + | Downstream to exons with higher exclusion  Upstream to exons with higher inclusion |
| QKI | COAD | 0.51 (p-value = 7.13e-05) | + | Downstream to exons with higher exclusion |
| QKI | LUAD | 0.41 (p-value = 1.77e-12) | + | Downstream to exons with higher exclusion  Upstream to exons with higher inclusion |
| QKI | KIRC | 1.32 (p-value = 0.036) | + | Downstream to exons with higher exclusion |
| QKI | PRAD | 0.71 (p-value = 0.021) | + | Downstream to exons with higher exclusion |
| MBNL1 | KIRC | 1.68 (p-value = 0.00011) |  |  |
| MBNL1 | COAD | 0.50 (p-value = 1.68e-09) | + | Downstream to exons with higher exclusion  Upstream to exons with higher inclusion |
| MBNL1 | BRCA | 0.58 (p-value = 2.27e-03) | + | All positions are enriched |
| MBNL1 | LUAD | 0.68 (p-value = 4.67e-03) | + | Downstream to exons with higher exclusion  Upstream to exons with higher inclusion |
| MBNL1 | PRAD | 0.70 (p-value = 0.015) |  |  |
| MBNL2 | BRCA | 0.55 (p-value = 0.00016) | + | All positions are enriched |
| MBNL2 | LIHC | 0.47 (p-value = 0.00066) | + | Upstream to exons with higher inclusion |
| MBNL2 | THCA | 0.71 (p-value = 0.0040) |  |  |
| MBNL2 | PRAD | 0.57 (p-value = 0.00012) |  |  |
| MBNL2 | LUAD | 0.73 (p-value = 0.019) | + | Upstream to exons with higher inclusion |
| PTBP1 | BRCA | 1.59 (p-value = 0.0054) | + | Upstream to exons with higher exclusion  Downstream to exons with higher inclusion |
| PTBP1 | COAD | 1.50 (p-value = 0.0015) |  |  |
| CELF2 | BRCA | 0.29 (p-value = 1.34e-12) | + | Upstream to exons with higher exclusion |
| CELF2 | PRAD | 0.55 (p-value = 4.32e-05) | + | Upstream to exons with higher exclusion |
| CELF2 | COAD | 0.63 (p-value = 0.00022) |  |  |
| CELF2 | KIRC | 1.99 (p-value = 1.63e-07) |  |  |
| CELF2 | LUAD | 0.44 (p-value = 3.43e-10) | + | Upstream to exons with higher inclusion |

Table S11. **Primers for RT-PCR validation**

| Chr | Exon start | Exon end | Gene | | Cancer types | | Inclusion (+)/ exclusion (-) in tumor | | F primer | | R primer | |  |
| --- | --- | --- | --- | --- | --- | --- | --- | --- | --- | --- | --- | --- | --- |
| Common exons to at least 5 cancer types | | | | | | | | |  | |  | |  |
| chr17 | 33997875 | 33997918 | AP2B1 | | LIHC, BRCA, LUAD, HNSC, COAD | | - | | AATGTGCCACAGGTGTCCTC | | TTCAGTCCACTGCTGACCAC | |  |
| chr6 | 131199243 | 131201347 | EPB41L2 | | BRCA, LUAD, PRAD, KIRC, COAD | | - | | CATTGCCGTGGTACAAGATG | | TTCGTCTTTCCCAACCTCTG | |  |
| chr3 | 13663274 | 13663416 | FBLN2 | | BRCA, LUAD, HNSC, COAD, THCA | | - | | CTCCTGTTTTCCCGGCTATG | | TGAGTGCCTTGTAGCAGTGG | |  |
| chr10 | 34661425 | 34661465 | PARD3 | | LIHC, BRCA, LUAD, KIRC, HNSC | | - | | CCCAAGAGGAAGTTGTTTCG | | CTGGTGCCATCAGGTGTAAG | |  |
| chr22 | 42564614 | 42564743 | TCF20 | | LIHC, BRCA, LUAD, KIRC, THCA | | - | | ACAACAAAGGCTGCTCCTTC | | TCCACCACCTTCTCATCTCC | |  |
| Potential splicing marker in one cancer type | | | | |  | |  | |  | |  | |  |
| chr4 | 85729486 | 85729571 | | WDFY3 | | BRCA | | + | | TCTTCCCCTCAAATACACAGC | | GGGGAGTTGAAACAGAATGATATG | |
| chr13 | 78141814 | 78141884 | | SCEL | | LUAD | | + | | TGAGAGAGATGTGCCAAAAGC | | TGGATGCTATGGTGTTGGAG | |
| chr3 | 152164492 | 152164547 | | MBNL1 | | LUAD, COAD, PRAD | | + | | CATTTGCAAGCCAAGATCAAG | | AGCAGGCCTCTTTGGTAATG | |

# Supplementary Methods

**Bioinformatic analysis**

Identification of enriched motifs

Significantly enriched motifs were identified for each cancer type by counting occurrences of all 4-6 possible k-mers within each cassette exon sequence, and 250 nt upstream and downstream within the flanking introns. Hypergeometric test was used to test the hypothesis that the prevalence of the k-mer in the cancer-altered cassette exons was not different from the highly expressed cassette exons that were not altered in this cancer-type. The test was applied to either cancer excluded or included cassette exons in each cancer type (determined by the average PSI change for each cancer type). False discovery rate (FDR) correction for multiple testing was applied.

In order to assess QKI and RBFOX motifs conservation, conservation measures were evaluated by PhastCons 100-way scores (38) , obtained from the UCSC database (39). For each motif, the conservation score was calculated as the mean of the phastCons scores for all the nucleotides within it, in all the downstream introns flanking altered cassette exons in cancer. Conservation score was calculated for the 20 nucleotides upstream and downstream to the motif for comparison. As a control, the same analysis was performed on all the downstream introns flanking the highly expressed cassette exons that were not significantly altered in any of the cancer types in our analysis.

Expression analysis

The DESeq package (40) in R was used for differential gene expression analysis on all normal and tumor samples. The gene annotations were obtained from UCSC gene tables from the UCSC database(39). The number of reads aligned to each gene was calculated using featureCounts (41) with the same alignment described above as input.

Knockdown comparison

Results of QKI and RBFOX2 knockdown experiments in SKOV3ip1 cell line on 93 alternative splicing events were obtained from (42). The knockdown shift was calculated as the delta between the mean PSI of two knockdown experiments and the mock transfected experiment.

Results of QKI knockdown and RBFOX2 ectopic expression RNA-seq experiments in BEAS2B and 293T cells respectively were obtained from (43, 44). For the QKI experiment, PSI levels were calculated using the reads supporting the relevant junctions for the statistically significant splicing events changed (Table S1, FDR <=0.05), and PSI change was calculated as the delta between the PSI of the knockdown experiments and the control. For the RBFOX2 experiment, PSI change was obtained from table S5 for the statistically significant splicing events changed (Table S5, FDR <=0.05).

Pearson correlation was calculated for each corresponding exon mean PSI shift in the cancer type examined (delta between tumor and normal samples) versus the PSI change in the relevant experiment only for cancer types that had at least 10 common events.

Pathway analysis

For each cancer type, functional analysis of the genes encoding differentially spliced cassette exons was performed with Ingenuity Pathways Analysis (Ingenuity Systems). Known biological relation and regulatory networks as well as other higher-order response pathways were analyzed using information in the Ingenuity Pathways Knowledge Base. Ingenuity functional analysis identified biological functions and/or diseases that were most significant. Annotations with a −log(*FDR*) > 3 calculated by a Fischer's exact test were selected for cluster analysis.

Survival analysis

Clinical data were obtained from the TCGA data portal. Survival was determined by “days to death” for uncensored patients, and “days to last follow-up” for right-censored patients. The association between splicing events that were significantly altered in at least five tumor types and survival was examined in these cancer types using the log rank test. High PSI change refers to PSI change greater than the 65^th^ percentile and low PSI change refers to PSI change lower than the 35^th^ percentile of all matched tumor–normal pairs for the examined splicing event. More than 20 matched samples were used for the examination.

**REFERENCES**

1. Zong,F.-Y., Fu,X., Wei,W.-J., Luo,Y.-G., Heiner,M., Cao,L.-J., Fang,Z., Fang,R., Lu,D., Ji,H., *et al.* (2014) The RNA-Binding Protein QKI Suppresses Cancer-Associated Aberrant Splicing. *PLoS Genet.*, **10**, e1004289.

2. Lovci,M.T., Ghanem,D., Marr,H., Arnold,J., Gee,S., Parra,M., Liang,T.Y., Stark,T.J., Gehman,L.T., Hoon,S., *et al.* (2013) Rbfox proteins regulate alternative mRNA splicing through evolutionarily conserved RNA bridges. *Nat. Struct. Mol. Biol.*, **20**, 1434–42.

3. Fiegen,D., Haeusler,L.-C., Blumenstein,L., Herbrand,U., Dvorsky,R., Vetter,I.R. and Ahmadian,M.R. (2004) Alternative splicing of Rac1 generates Rac1b, a self-activating GTPase. *J. Biol. Chem.*, **279**, 4743–9.

4. Matos,P., Collard,J.G. and Jordan,P. (2003) Tumor-related alternatively spliced Rac1b is not regulated by Rho-GDP dissociation inhibitors and exhibits selective downstream signaling. *J. Biol. Chem.*, **278**, 50442–8.

5. Misquitta-Ali,C.M., Cheng,E., O’Hanlon,D., Liu,N., McGlade,C.J., Tsao,M.S. and Blencowe,B.J. (2011) Global profiling and molecular characterization of alternative splicing events misregulated in lung cancer. *Mol. Cell. Biol.*, **31**, 138–50.

6. Hervé,M.-A., Buteau-Lozano,H., Mourah,S., Calvo,F. and Perrot-Applanat,M. (2005) VEGF189 stimulates endothelial cells proliferation and migration in vitro and up-regulates the expression of Flk-1/KDR mRNA. *Exp. Cell Res.*, **309**, 24–31.

7. Li,C., Kato,M., Shiue,L., Shively,J.E., Ares,M. and Lin,R.-J. (2006) Cell type and culture condition-dependent alternative splicing in human breast cancer cells revealed by splicing-sensitive microarrays. *Cancer Res.*, **66**, 1990–9.

8. Gardina,P.J., Clark,T.A., Shimada,B., Staples,M.K., Yang,Q., Veitch,J., Schweitzer,A., Awad,T., Sugnet,C., Dee,S., *et al.* (2006) Alternative splicing and differential gene expression in colon cancer detected by a whole genome exon array. *BMC Genomics*, **7**, 325.

9. Brinkman,B.M.N. (2004) Splice variants as cancer biomarkers. *Clin. Biochem.*, **37**, 584–594.

10. Ge,K., DuHadaway,J., Du,W., Herlyn,M., Rodeck,U. and Prendergast,G.C. (1999) Mechanism for elimination of a tumor suppressor: Aberrant splicing of a brain-specific exon causes loss of function of Bin1 in melanoma. *Proc. Natl. Acad. Sci.*, **96**, 9689–9694.

11. Karni,R., de Stanchina,E., Lowe,S.W., Sinha,R., Mu,D. and Krainer,A.R. (2007) The gene encoding the splicing factor SF2/ASF is a proto-oncogene. *Nat. Struct. Mol. Biol.*, **14**, 185–93.

12. Xi,L., Feber,A., Gupta,V., Wu,M., Bergemann,A.D., Landreneau,R.J., Litle,V.R., Pennathur,A., Luketich,J.D. and Godfrey,T.E. (2008) Whole genome exon arrays identify differential expression of alternatively spliced, cancer-related genes in lung cancer. *Nucleic Acids Res.*, **36**, 6535–47.

13. Gaur,S., Shively,J.E., Yen,Y. and Gaur,R.K. (2008) Altered splicing of CEACAM1 in breast cancer: identification of regulatory sequences that control splicing of CEACAM1 into long or short cytoplasmic domain isoforms. *Mol. Cancer*, **7**, 46.

14. Brauer,P.M., Zheng,Y., Evans,M.D., Dominguez-Brauer,C., Peehl,D.M. and Tyner,A.L. (2011) The alternative splice variant of protein tyrosine kinase 6 negatively regulates growth and enhances PTK6-mediated inhibition of β-catenin. *PLoS One*, **6**, e14789.

15. Yamaguchi,F., Saya,H., Bruner,J.M. and Morrison,R.S. (1994) Differential expression of two fibroblast growth factor-receptor genes is associated with malignant progression in human astrocytomas. *Proc. Natl. Acad. Sci.*, **91**, 484–488.

16. Kobrin,M.S., Yamanaka,Y., Friess,H., Lopez,M.E. and Korc,M. (1993) Aberrant Expression of Type I Fibroblast Growth Factor Receptor in Human Pancreatic Adenocarcinomas Advances in Brief Aberrant Expression of Type I Fibroblast Growth Factor Receptor in Human Pancreatic Adenocarcinomas. *Cancer Res.*

17. Frasca,F., Pandini,G., Scalia,P., Sciacca,L., Mineo,R., Costantino,A., Goldfine,I.D., Belfiore,A. and Vigneri,R. (1999) Insulin Receptor Isoform A, a Newly Recognized, High-Affinity Insulin-Like Growth Factor II Receptor in Fetal and Cancer Cells. *Mol. Cell. Biol.*, **19**, 3278–3288.

18. Huang,R., Xing,Z., Luan,Z., Wu,T., Wu,X. and Hu,G. (2003) A Specific Splicing Variant of SVH, a Novel Human Armadillo Repeat Protein, Is Up-Regulated in Hepatocellular Carcinomas. *Cancer Res.*, **63**, 3775–3782.

19. Wang,L., Duke,L., Zhang,P.S., Arlinghaus,R.B., Symmans,W.F., Sahin,A., Mendez,R. and Dai,J. Le (2003) Alternative Splicing Disrupts a Nuclear Localization Signal in Spleen Tyrosine Kinase That Is Required for Invasion Suppression in Breast Cancer. *Cancer Res.*, **63**, 4724–4730.

20. Hindermann,W., Berndt,A., Borsi,L., Luo,X., Hyckel,P., Katenkamp,D. and Kosmehl,H. (1999) Synthesis and protein distribution of the unspliced large tenascin-C isoform in oral squamous cell carcinoma. *J. Pathol.*, **189**, 475–80.

21. Dueck,M., Riedl,S., Hinz,U., Tandara,A., Möller,P., Herfarth,C. and Faissner,A. (1999) Detection of tenascin-C isoforms in colorectal mucosa, ulcerative colitis, carcinomas and liver metastases. *Int. J. Cancer*, **82**, 477–83.

22. Borsi,L., Carnemolla,B., Nicolò,G., Spina,B., Tanara,G. and Zardi,L. (1992) Expression of different tenascin isoforms in normal, hyperplastic and neoplastic human breast tissues. *Int. J. Cancer*, **52**, 688–692.

23. Cheung,H.C., Baggerly,K.A., Tsavachidis,S., Bachinski,L.L., Neubauer,V.L., Nixon,T.J., Aldape,K.D., Cote,G.J. and Krahe,R. (2008) Global analysis of aberrant pre-mRNA splicing in glioblastoma using exon expression arrays. *BMC Genomics*, **9**, 216.

24. Pujuguet,P., Hammann,A., Moutet,M., Samuel,J.L., Martin,F. and Martin,M. (1996) Expression of fibronectin ED-A+ and ED-B+ isoforms by human and experimental colorectal cancer. Contribution of cancer cells and tumor-associated myofibroblasts. *Am. J. Pathol.*, **148**, 579–92.

25. Matsui,S., Takahashi,T., Oyanagi,Y., Takahashi,S., Boku,S., Takahashi,K., Furukawa,K., Arai,F. and Asakura,H. (1997) Expression, localization and alternative splicing pattern of fibronectin messenger RNA in fibrotic human liver and hepatocellular carcinoma. *J. Hepatol.*, **27**, 843–853.

26. Dutertre,M., Lacroix-Triki,M., Driouch,K., de la Grange,P., Gratadou,L., Beck,S., Millevoi,S., Tazi,J., Lidereau,R., Vagner,S., *et al.* (2010) Exon-based clustering of murine breast tumor transcriptomes reveals alternative exons whose expression is associated with metastasis. *Cancer Res.*, **70**, 896–905.

27. Mochizuki,H., Nishi,T., Bruner,J.M., Lee,P.S.Y., Levin,V.A. and Saya,H. (1992) Alternative splicing of neurofibromatosis type 1 gene transcript in malignant brain tumors: PCR analysis of frozen-section mRNA. *Mol. Carcinog.*, **6**, 83–87.

28. Thorsen,K., Sørensen,K.D., Brems-Eskildsen,A.S., Modin,C., Gaustadnes,M., Hein,A.-M.K., Kruhøffer,M., Laurberg,S., Borre,M., Wang,K., *et al.* (2008) Alternative splicing in colon, bladder, and prostate cancer identified by exon array analysis. *Mol. Cell. Proteomics*, **7**, 1214–1224.

29. Baird,B.N., Schliekelman,M.J., Ahn,Y.H., Chen,Y., Roybal,J.D., Gill,B.J., Mishra,D.K., Erez,B., O’Reilly,M., Yang,Y., *et al.* (2013) Fibulin-2 Is a Driver of Malignant Progression in Lung Adenocarcinoma. *PLoS One*, **8**.

30. Law,E.W.L., Cheung,A.K.L., Kashuba,V.I., Pavlova,T. V, Zabarovsky,E.R., Lung,H.L., Cheng,Y., Chua,D., Lai-wan Kwong,D., Tsao,S.W., *et al.* (2012) Anti-angiogenic and tumor-suppressive roles of candidate tumor-suppressor gene, Fibulin-2, in nasopharyngeal carcinoma. *Oncogene*, **31**, 728–738.

31. Tomimaru,Y., Xu,C.Q., Nambotin,S.B., Yan,T., Wands,J.R. and Kim,M. (2013) Loss of exon 4 in a human T-cell factor-4 isoform promotes hepatic tumourigenicity. *Liver Int.*, **33**, 1536–48.

32. Okumura,M., Kondo,S., Ogata,M., Kanemoto,S., Murakami,T., Yanagida,K., Saito,A. and Imaizumi,K. (2005) Candidates for tumor-specific alternative splicing. *Biochem. Biophys. Res. Commun.*, **334**, 23–9.

33. Ponthier,J.L., Schluepen,C., Chen,W., Lersch,R.A., Gee,S.L., Hou,V.C., Lo,A.J., Short,S.A., Chasis,J.A., Winkelmann,J.C., *et al.* (2006) Fox-2 splicing factor binds to a conserved intron motif to promote inclusion of protein 4.1R alternative exon 16. *J. Biol. Chem.*, **281**, 12468–12474.

34. Marquis,J., Paillard,L., Audic,Y., Cosson,B., Danos,O., Le Bec,C. and Osborne,H.B. (2006) CUG-BP1/CELF1 requires UGU-rich sequences for high-affinity binding. *Biochem. J.*, **400**, 291–301.

35. Faustino,N.A. and Cooper,T.A. (2005) Identification of putative new splicing targets for ETR-3 using sequences identified by systematic evolution of ligands by exponential enrichment. *Mol. Cell. Biol.*, **25**, 879–887.

36. Goers,E.S., Purcell,J., Voelker,R.B., Gates,D.P. and Berglund,J.A. (2010) MBNL1 binds GC motifs embedded in pyrimidines to regulate alternative splicing. *Nucleic Acids Res.*, **38**, 2467–2484.

37. Auweter,S.D., Fasan,R., Reymond,L., Underwood,J.G., Black,D.L., Pitsch,S. and Allain,F.H.-T. (2006) Molecular basis of RNA recognition by the human alternative splicing factor Fox-1. *EMBO J.*, **25**, 163–173.

38. Llorian,M., Schwartz,S., Clark,T.A., Hollander,D., Tan,L.-Y., Spellman,R., Gordon,A., Schweitzer,A.C., de la Grange,P., Ast,G., *et al.* (2010) Position-dependent alternative splicing activity revealed by global profiling of alternative splicing events regulated by PTB. *Nat. Struct. Mol. Biol.*, **17**, 1114–1123.

39. Xue,Y., Zhou,Y., Wu,T., Zhu,T., Ji,X., Kwon,Y.S., Zhang,C., Yeo,G., Black,D.L., Sun,H., *et al.* (2009) Genome-wide Analysis of PTB-RNA Interactions Reveals a Strategy Used by the General Splicing Repressor to Modulate Exon Inclusion or Skipping. *Mol. Cell*, **36**, 996–1006.

40. Galarneau,A. and Richard,S. (2005) Target RNA motif and target mRNAs of the Quaking STAR protein. *Nat. Struct. Mol. Biol.*, **12**, 691–8.
